# The Impact of Multifunctional Genes on "Guilt by Association" Analysis

**DOI:** 10.1371/journal.pone.0017258

**Published:** 2011-02-18

**Authors:** Jesse Gillis, Paul Pavlidis

**Affiliations:** 1 Centre for High-Throughput Biology, Department of Psychiatry, University of British Columbia, Vancouver, British Columbia, Canada; 2 Michael Smith Laboratories, University of British Columbia, Vancouver, British Columbia, Canada; Johns Hopkins University, United States of America

## Abstract

Many previous studies have shown that by using variants of “guilt-by-association”, gene function predictions can be made with very high statistical confidence. In these studies, it is assumed that the “associations” in the data (e.g., protein interaction partners) of a gene are necessary in establishing “guilt”. In this paper we show that multifunctionality, rather than association, is a primary driver of gene function prediction. We first show that knowledge of the degree of multifunctionality alone can produce astonishingly strong performance when used as a predictor of gene function. We then demonstrate how multifunctionality is encoded in gene interaction data (such as protein interactions and coexpression networks) and how this can feed forward into gene function prediction algorithms. We find that high-quality gene function predictions can be made using data that possesses no information on which gene interacts with which. By examining a wide range of networks from mouse, human and yeast, as well as multiple prediction methods and evaluation metrics, we provide evidence that this problem is pervasive and does not reflect the failings of any particular algorithm or data type. We propose computational controls that can be used to provide more meaningful control when estimating gene function prediction performance. We suggest that this source of bias due to multifunctionality is important to control for, with widespread implications for the interpretation of genomics studies.

## Introduction

Understanding the function of genes is one of the central challenges of biology [1], [2], [3]. Characterizing gene function is complex, in part because biological functions involve the integrated activities of many genes. The same gene may have different functions depending on context, which is in turn be defined partly by the presence of other gene products. For example, the tumor suppressor TP53 has different functions depending on its interaction partners (e.g. [4], [5], [6], [7]). In this paper we are concerned with issues surrounding “multifunctionality” at the molecular level. While we define “multifunctionality” precisely below, we intend the term to mean approximately “the number of functions a gene is involved in”. We are interested in how multifunctionality impacts the interpretation of experiments, especially from the standpoint of computational analyses that are applied to large high-throughput data sets such as expression profiling and proteomics surveys. In particular, we take a close look at how the degree of multifunctionality (whether it is known or not) interacts with the computational assignment of functions to genes. This seemingly esoteric issue turns out to have surprisingly deep implications in how high-throughput data sets are interpreted.

Despite the obvious importance of understanding gene function, multifunctionality has received surprisingly little attention in the functional genomics literature. There appears to be little consensus on the definition of “multifunctionality”. Previous work has considered attributes of genes which, intuitively, might be related to multifunctionality: pleiotropy, promiscuity, and hub-ness, but these are rarely discussed in the context of multifunctionality. While closest to multifunctionality in definition, pleiotropy (the ability of a gene to influence multiple phenotypic traits) is not typically used to refer exclusively to molecular traits and is usually defined with reference to the effect of mutation on phenotype. In contrast, we will use “multifunctional” to refer to genes possessing multiple molecular functions, each of which can be characterized by the set of genes (or their products) inferred to be interacting in a particular biological context. Thus, pleiotropy is both usually further downstream phenotypically than multifunctionality and defined with reference to the effects of allelic variation as opposed to observed or inferred molecular interaction. Pleiotropic genes are suggested to tend to be conserved [8], modular [9], involved in more biological processes [10], and more commonly interacting [11]. However, many of these characterizations have been theoretical [12], with experimental evidence being mixed [13], [14], [15]. Pleiotropy can be formally assessed by the effect of mutation on phenotypic profile [13], but the determination of a pleiotropic gene will depend on the functional categories chosen (or the contexts over which phenotypic profile is measured). Similarly, hub genes and promiscuous genes may be defined as genes which possess many interactions (e.g., [16], [17]), though there is no principled basis for choosing the threshold as to how many interactions is “many”. Hubs tend to be essential ([18], [19]), conserved ([20], [21]) (or, alternatively, intrinsically disordered and non-conserved [22], and abundant[23]. The high connectivity of hubs (along with conservation) is generally taken to reflect biological “importance”, although this is not fully resolved [24]. In contrast, the term “promiscuous proteins” is usually used to refer to “sticky” interactors whose interactions are “non-specific” and due to analysis artifacts [16]. Recently promiscuity has been considered as potentially functional [25], but this appears to be a minority view. One question embodied in the terminological distinction between promiscuous proteins (non-specific) and hub genes (functional) is the specificity of function itself. A distinction between promiscuity and “hub-ness”, for example, may be that (some) hubs are strongly/specifically involved in many functions whereas promiscuous proteins are only weakly/uncertainly involved in many functions [26].We propose that the cloudiness surrounding these issues (e.g., [27]) can be in part resolved by carefully considering what is meant by “multifunctionality”, and using the resulting precise definition to analyze gene networks.

An important aspect of the work we present is the general method used for describing and assessing function using computational techniques. Three things are required. First, genes must be classified into functions using some scheme; the most popular such repository of functional information is the Gene Ontology [28]. Second, an algorithm is needed to assign functions. Gene function prediction algorithms attempt to determine candidate genes for a functional group (e.g., GO group) using the properties of the existing genes in the set. These properties form the third requirement for function prediction. Typically, the properties of genes used in gene function prediction are represented as a network (graph) of gene associations defined by any of a number of methods, including protein interactions [29], [30], [31], [32], RNA coexpression [3], [33], [34] and genetic interactions [35], [36], [37]. More broadly, these networks encode either interactions (e.g., protein interaction, genetic interaction) or similar profiles across functional contexts (e.g., coexpression, phylogenetic profile), both of which imply shared function. This is partly expressed in the principle of “guilt by association” which states that genes which are associated (i.e., interaction or profile) are more likely to share function [38]. Thus one chooses a target gene group of interest and uses “guilt by association” of one form or another to decide which other genes in the network should be assigned the same “function”. Such approaches have been shown to be broadly applicable, with a high degree of success in predicting gene function in cross-validation settings [39], [40], [41], [42], [43]. In one study [43], essentially all GO categories were at least somewhat learnable from expression data.

A main point of the current paper is that contrary to assumptions inherent in previous studies, most computational predictions of function are driven by the presence of multifunctionality. This leads us to a very different interpretation of guilt-by-association than that which is usually offered. It is therefore of interest to us that since the guilt-by-association approach was first articulated, there have been a variety of efforts to characterize difficulties with it. In particular, it is recognized that false positive predictions are a problem. One issue is false positives in the original network [31], [44], [45] – in other words, promiscuity that leads to false associations among genes and thus among genes and functions. A tactic that has been applied to coexpression analysis is to retain an edge in the network only if the given edge is among the highest scoring candidate edges for both genes. We referred to this as the “top overlap” method. [39], [45], [46], [47] use variations on this approach. Another class of problem has to do with the choice of the “negative” control group of genes used in the algorithm for prediction, leading to inflated performance measures [48], [49], [50], [51]. We show that approaches to remedy these problems have other effects on prediction of function which can be understood by how the methods interact with multifunctionality.

Another key concept for our work is node degree (the number of connections per gene in a network) and how it relates to function. Node degree is a central property of gene networks [52] and in fact can be used to help predict the interactions of genes[53]. Because genes function in a large part by interacting with other genes, one might expect multifunctional genes to exhibit higher node degree [54]. The possibility of a correlation between multifunctionality and node degree has been previously examined as part of the general interest in characterizing properties associated with node degree, although these results have been mixed [10], [11], [14], [22]. In this paper we show that node degree is unambiguously linked to multifunctionality even in cases where the two properties are uncorrelated – and further that this has strong implications in how functions get associated with genes.

Using a range of data, algorithms, and analytical tools, in this paper we provide strong evidence that multifunctionality a key factor in explaining the results of gene function prediction analyses. This is important because there is an unstated assumption in the literature that gene function predictions are “specific” to the function in question. The truth is that most gene function predictions are driven by the tendency of algorithms to simply assign more functions to genes which are already multifunctional. Indeed, we believe that there are no computational gene function assignments that can be shown to preclude the possibility they reflect only underlying multifunctionality. This is in spite of the fact that some predictions go on to be confirmed by laboratory methods: a correct prediction does not mean the predictive method is working the way one thinks it does. We propose that these concepts are important not just to computational gene function prediction, but highly relevant to the interpretation of all biological studies that try to probe gene function.

## Results

### Defining multifunctionality and predicting without data

Using GO as our source of functional annotations, we define the multifunctionality of a single gene as
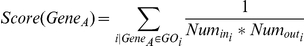
where Num_in_i_ is the number of genes within GO group i, and Num_out_i_ is the number of genes outside GO group i. If we ignore the weighting by the size of the groups, this score is simply the number of GO terms a gene has. The weighting has the effect of counting membership in a GO group by how much the gene contributes to that GO group. Weighting by Num_in has the effect of giving a gene which, e.g., is one out of five in a GO group a contribution of 1/5. Weighting by Num_out provides a corresponding weighting to genes not within the GO group; that is, being the only gene outside a large GO group subtracts as much from that one gene's score as being the only gene within a GO group would add to another gene's score. The particular form of this definition was not chosen arbitrarily, as we now explain.

**Figure 1:**
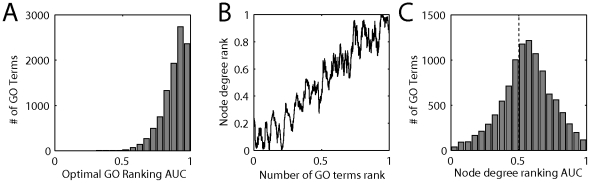
A single ranking of genes can predict GO group membership. A) The distribution of AUCs yielded by the “optimal gene ranking” for 10127 GO groups of size 20–1000. This list is similar to ordering genes by the number of GO categories to which they belong. B) The number of GO groups per gene is correlated with node degree. The number of coexpression partners (in those genes with at least one coexpression partner) is plotted as a function of the number of GO categories (binned) to which a gene belongs. Values were ranked and the bin size was set to 500 genes (to capture the consistent trend in the weak rank correlation of 0.28). C) Node degree alone can predict GO group membership. The histogram of AUCs across all GO groups which can be obtained using a single list constructed from number of coexpression partners. The median is well above 0.5.

We arrived at our definition of multifunctionality by considering that the greater the multifunctionality of a gene, the greater the degree to which it should be a good candidate for having *any* function (averaged over all functions). Thus, a single ranked list of genes which best captures candidacy across all functions is equivalent to a list of genes ranked by multifunctionality. Intuitively, if one is forced to choose a single ranking, the gene with the most GO annotations could be predicted as being in *all* GO categories. This is because if one gene is in 100 GO categories (high multifunctionality), and another is in only one (low multifunctionality), by placing the former gene ahead of the latter gene in a fixed ranking, we make a correct prediction more often across all GO categories. Because the ranking of genes is optimized in this way, we expect that when we use it as a “predictor” of GO category membership, we should get values of the AUC of over 0.5 for many GO terms. We were nonetheless surprised that this single ranking of genes gives a mean AUC of 0.90 across all GO terms tested (Figure 1A). By itself, this is clearly not a meaningful result for “gene function prediction” since the ranking is obtained in a “circular” way by optimizing for GO. A main point of this paper is that there are properties of real data that are correlated with this optimal ranking, and this fact can explain much of the apparent learnability of gene function by “guilt-by-association”.

The definition above is the optimal list (see Data S1, Section 1) when the optimization is for the area under the curve (AUC) of the receiver-operator curve (ROC).

Small differences in AUC can hide larger differences in other measures such as positive predictive value (PPV). Maximizing AUC provides a particularly intuitive form for the multifunctionality scores (number of functions weighted by involvement) as well as simple analytic calculation of the optimal list, but the same principle can be applied to other metrics. The AUC has the added benefit of being widely used and not dependent on choosing a specific threshold. Using a different metric would suggest using a different optimal list. In later sections we show that our conclusions are not sensitive to the choice of performance measure.

It is naturally of interest to know which genes are most multifunctional. As ranked by our definition, the most multifunctional human genes include a mixture of genes that most biologists would recognize immediately as "centrally important" (e.g. tumor necrosis factor (TNF), transforming growth factor beta 1 (TGFB1)) and others that might seem more unexpected (e.g. forkhead box J1 (FOXJ1), ATPase, Cu^++^ transporting, alpha polypeptide (ATP7A); the full lists are available as Data S1). We leave as a topic for future work the question of whether these top-ranked genes really are especially multifunctional, or whether they merely appear to be, due to biases in research into gene function or of patterns of annotation. In addition to providing a single ranking of genes by multifunctionality, we also obtain a ranking of GO groups by how well they are predicted by this ranking (Data S1). Highly-ranked GO groups are the “most multifunctional” – the genes they contain tend to be highly multifunctional.

Thus far we have merely provided a novel definition of multifunctionality and shown how it can yield surprisingly strong performance when used for functional prediction. As mentioned above, while this exposes some important features about the distribution of GO annotations amongst genes and gives insight into which genes are most “multifunctional”, by itself it has no implication for gene function prediction because it uses GO in its construction (it is obviously “overfit”; we are not proposing this ranking is of any utility for gene function prediction). We now show that the ranking of genes by multifunctionality is consistently reflected in real data. The basic implication is that gene function predictions based on such data can be attributed to multifunctionality.

### The relationship between multifunctionality and network node degree

We hypothesized that multifunctionality plays a role in the prediction of gene function from genomics data. In particular, we wished to examine whether multifunctionality is reflected in other properties of genes which are used in data interpretation. This is potentially important because it is usually assumed that when genes are assigned a function, it is due to either a valid prediction or a false positive due to “promiscuity” or other issues with the data. We suspected that in fact multifunctionality can explain a substantial amount of the way functions are assigned. How could this happen? As we showed in Figure 1A, ranking genes by multifunctionality would be a good way to get good performance from a gene function prediction algorithm. Thus, if the data used for prediction is in some way a proxy for (or correlated with) multifunctionality, and the algorithm used for classification can exploit this, very good prediction performance can result. Put another way, algorithms which assign new functions to genes which are already highly multifunctional will, on average, be rewarded by appearing to yield good performance. This is true no matter what measure of performance is used, though as mentioned above the optimal ranking will be somewhat different for different performance measures.

Because gene function prediction uses the structure of gene networks (or association matrices), it is natural to consider how those networks are generated and how this relates to multifunctionality. A key statistic about networks is the node degree of each gene – how many connections each gene has. Because we define each function at the molecular level by the set of genes interacting in a particular context, it might be expected that a greater number of interaction partners overall (higher node degree) would reflect membership in a greater number of sets of genes (higher multifunctionality); this is the hub view of high node degree. Alternatively, functional studies might not translate well to interaction studies, or a higher node degree could represent only a less specifically defined functional role for the gene for no greater number of functions; this is the promiscuous protein view of high node degree.

In the following we consider a model to assess the specificity of functional information in protein interaction networks (and find that node degree ranks genes by their probability of random interaction). Previous workers have noted that in protein interaction networks, the probability of two genes being associated in a network is strongly correlated with the product of their individual node degrees (the number of connections they have) [53]. It has further been shown that protein interaction networks can be surprisingly accurately reconstructed by a model in which each gene *i* has an inherent and fixed probability *p_i_* of being an interactor, without any specificity in the interactions; in our model the probability of an interaction is determined only by the product of these probabilities.

An “interaction” matrix generated from this model takes the form *p_i_p_j_* for each pair of genes *i* and *j* – the independent product of probabilities representing the probability of both proteins appearing in the test and thereby their being labeled as associated. If we treat a real interaction matrix as if it were generated from the model, we can reconstruct the ranking of the original values for the *p's* by noting that the sums across the columns of the matrix are
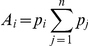



Because the sum forming the multiplicand is a constant, this product is proportional to the value of *p_i_*, so the result is the desired ranking. With a real interaction matrix, we compute A simply as the (weighted) sum of each row of the matrix. If the original network is unweighted, A is essentially equivalent to the node degree. Under this null model, node degree is more than just an interesting statistic about a gene; it explains the structure of networks.

Our interest in node degree is spurred by the idea that node degree might be strongly related to multifunctionality. As mentioned in the introduction, this seems intuitive and has some support in the literature. Here we show that there is an unequivocal relationship between node degree and multifunctionality, in a wide range of networks.

We first computed node degree for a human gene coexpression network and compared it to the ranking provided by the degree of annotations (number of GO terms) (Figure 1B). The rank correlation is modest (0.28) but statistically significant (p<0.001). Importantly, the effect is distributed across the entire range of node degrees – it is not just a property of genes with higher node degrees. This general relationship held for a variety of other networks we examined (Table 1), to varying degrees. Thus there is a relationship between multifunctionality and node degree, but by this measure the effect appears modest.

**Table 1:** Gene function prediction performance.

Matrix	Gene sets	Algorithm	Score (AUC or CCR)	Node degree score	Correlation
PPIN	GO	GeneMANIA	0.70	0.63	0.95
PPIN	GO	SVM	0.60	0.64	0.65
PPIN	KEGG	GeneMANIA	0.73	0.65	0.97
PPIN	KEGG	SVM	0.66	0.66	0.85
Coexpression	GO	GeneMANIA	0.59	0.54	0.83
Coexpression	GO	SVM	0.53	0.55	0.36
Coexpression	KEGG	GeneMANIA	0.63	0.56	0.81
Threshold Coexpression	GO	GeneMANIA	0.55	0.52	0.96

Table 1: Each combination of data, method, and gene set is shown along with its performance. The scores are ROC areas for GeneMANIA and correct classification rates for SVM. The performance of the node degree vector for each set is also shown, along with the correlation between the two sets of scores.

As hinted above, the results shown in Figures 1A and 1B suggest that simply ranking genes by how much coexpression they have (regardless of with which other gene) would yield good results in classification tasks because it would approximately rank genes by their multifunctionality. In this approach, the gene with the highest node degree (most widely coexpressed) is predicted as the best candidate for membership in *all* GO categories, and the gene with the second-highest node degree is the next best candidate, and so on. The results of doing this for coexpression data are shown in Figure 1C. The mean ROC is 0.58, which is significantly different from 0.5 (p<0.01, permutation test), and there are many GO terms for which performance is quite good (over 0.70). We performed a similar analysis on several other networks (Table 1), and the performance is consistently significant (p<0.01). The performance is particularly strong in the case of the human protein-protein interaction network (Table 1). Clearly these prediction performance results are an artifact, and are due to interactions between the input data and multifunctionality.

The results in Figure 1B and Table 1 show that while there is a detectable relationship between node degree and multifunctionality, the actual correlation between the rankings is modest and varies from data set to data set. However, when we look at the impact on the ability to predict gene function from node degree alone, the variance is almost entirely explainable by multifunctionality. To help show this, we assessed the performance of node degree at predicting gene function in forty-seven human coexpression networks. As expected, performance varies from data set to data set; these values essentially expand the data in Table 1. Overall, the mean AUC we obtain with a given network using node degree alone to rank genes is highly predicted by the degree to which the network's node degree ranking correlates with multifunctionality (Spearman correlation  =  0.96). Thus node degree performance is a proxy for multifunctionality in determining gene interactions. In subsequent sections, we will see more clearly that node degree performance is central to determining overall performance in gene function prediction from networks, so the high correlation between node degree performance and the degree to which node degree reflects multifunctionality is critical.

Thus far we have measured multifunctionality using the Gene Ontology, which is arguably the best single source of gene annotations. One could ask whether the same ranking generalizes for other groupings of genes one might want to predict. As shown in Table 2, the GO-based multifunctionality ranking provides very good predictions for sets of candidate disease genes identified in genetics studies, including Alzheimer's disease [55], schizophrenia [56], Parkinson's disease [57] and autism [58]. Indeed, across 4069 sets of disease genes from OMIM [59], the average ROC is 0.76 (Figure S1). To give a point of reference, the multifunctionality ranking performs better than a sophisticated algorithm, GeneMANIA [39], using protein interaction data to make predictions on these same groups (Table 2). This finding suggests biases in how these candidate gene groups are established, how association with disease indirectly influences the GO annotations a gene receives, and/or the degree to which multifunctional genes contribute to disease.

**Table 2:** Disease candidate gene prediction.

	Alzheimer's	Schizophrenia	Parkinson's	Autism	OMIM
Optimum List from GO (ROC AUC)	0.78	0.73	0.73	0.71	0.76+/−0.22
Machine Learning (ROC AUC)	0.74	0.69	0.71	0.67	–

Table 2: We predicted disease genes using the optimal gene ranking based on GO and then validated using disease gene candidate sets (top). These ROC results compare favorably to predicting candidate genes using a strongly performing protein interaction network and a powerful gene function prediction algorithm (bottom). OMIM disease gene sets are too small for cross-validation using a gene function prediction algorithm.

In this section we have shown that using node degree alone to predict gene function works strikingly well and that numerous real-life rankings of genes seem to reflect multifunctionality more than any more specific biological principle. But because node degree is not actually what is used by biologists to predict function, one might still ask whether “real” analyses are independent of these effects. We address this issue in the next section.

### Do gene networks provide new information?

While the above results show that simply ranking genes by node degree (and thus by multifunctionality) provides some predictability of gene function, it is still possible that a “real” analysis of the original network with a sophisticated machine learning algorithm does much better and that multifunctionality plays only a small role in real studies. Unfortunately we find this is not the case. Earlier we mentioned the finding that node degree has been shown to explain much of the structure of protein interaction networks. We now show that node degree (and thus multifunctionality) underlies a large fraction of the performance of gene function prediction methods. In fact is it impossible to say for certain that any gene function prediction is not simply due to the influence of multifunctionality.

**Figure 2:**
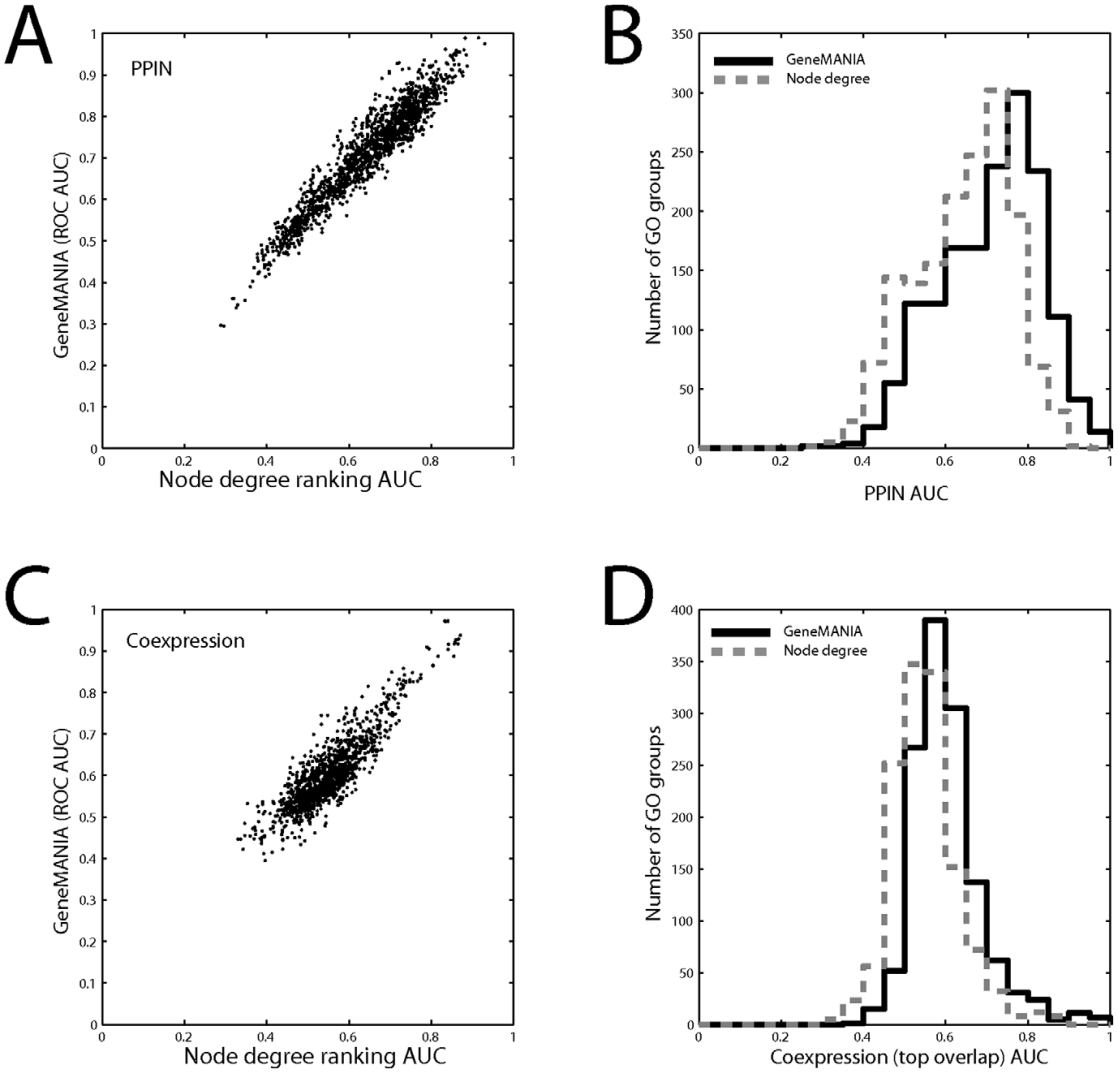
Node degree performance is highly correlated with prediction from the original data. A) The performance of a single list of genes ranked by node degree of a protein interaction network is compared to the performance using GeneMANIA for the interaction network across all GO groups of size 20–1000. B) The protein interaction node degree vector gives comparable performance to the true network performance. C) The performance of node degree using coexpression data. D) The coexpression node degree vector gives comparable performance to the true network performance. Only data in which some interaction is present within a given GO group – the learnable groups - is shown.

As a first indication of this problem, we found that the ability to predict a gene function group based on node degree performance is strongly predictive of how well that group will be predicted in the “real” analysis (Table 1, Figure 2A and 2C). That is, node degree generates its best prediction performance in the same gene groups as a real analysis. In fact, the correlation understates this trend, since scatter around an AUC of 0.5 is expected. Second, predictions from node degree are surprisingly good compared to the “real” analysis (Figure 2B and D). For example, while average “real” prediction performance of GO groups is high when using protein interactions (mean AUC 0.7), the node degree ranking mean AUC is 0.63 – far above the 0.5 expected by chance. Coexpression yielded very similar results (Table 1). Thus, association matrices reflecting strong multifunctionality produce better performance, and particularly the performance that could be predicted by multifunctionality alone (without knowledge of specific gene-gene associations).

The histograms in Figure 2B and D show another important feature of node degree performance. Not only is node degree only slightly worse than real performance, the distributions have similar variances. This suggests that if the statistical significance of the predictions made from the real data were calibrated by the performance from node degree, the results would be drastically different from the more usual approach of using a permuted matrix. In other words, not only is “real” performance largely similar to multifunctionality performance, multifunctionality explains much of the variation for one group compared to others.

While node degree ranking is surprisingly powerful for predicting gene function, it is still not clear from the above analysis the extent to which node degree underlies the performance obtained when network structure is used. To quantify how much is actually gained by using the network and show the manner in which multifunctionality can affect network structure through node degree, we constructed networks entirely according to the model proposed earlier (in which a gene's appearance in a given test is taken to be due to a higher prevalence, *p_i_*). As earlier, our interaction matrix takes the form of a self-outer product, and as shown in the supplement (Data S1, Section 2), the vector which provides the best approximation (in the least-squares sense) of the original association matrix under this constraint is the node degree vector. This means that the best approximation (under these conditions) is a network constructed from our proxy for multifunctionality. We call the network formed from the self outer product of the node degree vector an “individual property network” or IPN, since it uses only information about individual genes (i.e., node degree), not relationships between genes. This network lacks any meaningful association information but can be analyzed using the same algorithms as the original network (see Figure S2 for a schematic of IPN construction). Thus, IPNs reflect associations predicted from multifunctionality alone, that genes with many functions are more likely to interact, and if these interactions show gene function prediction performance, it can be ascribed to multifunctionality alone.

**Figure 3:**
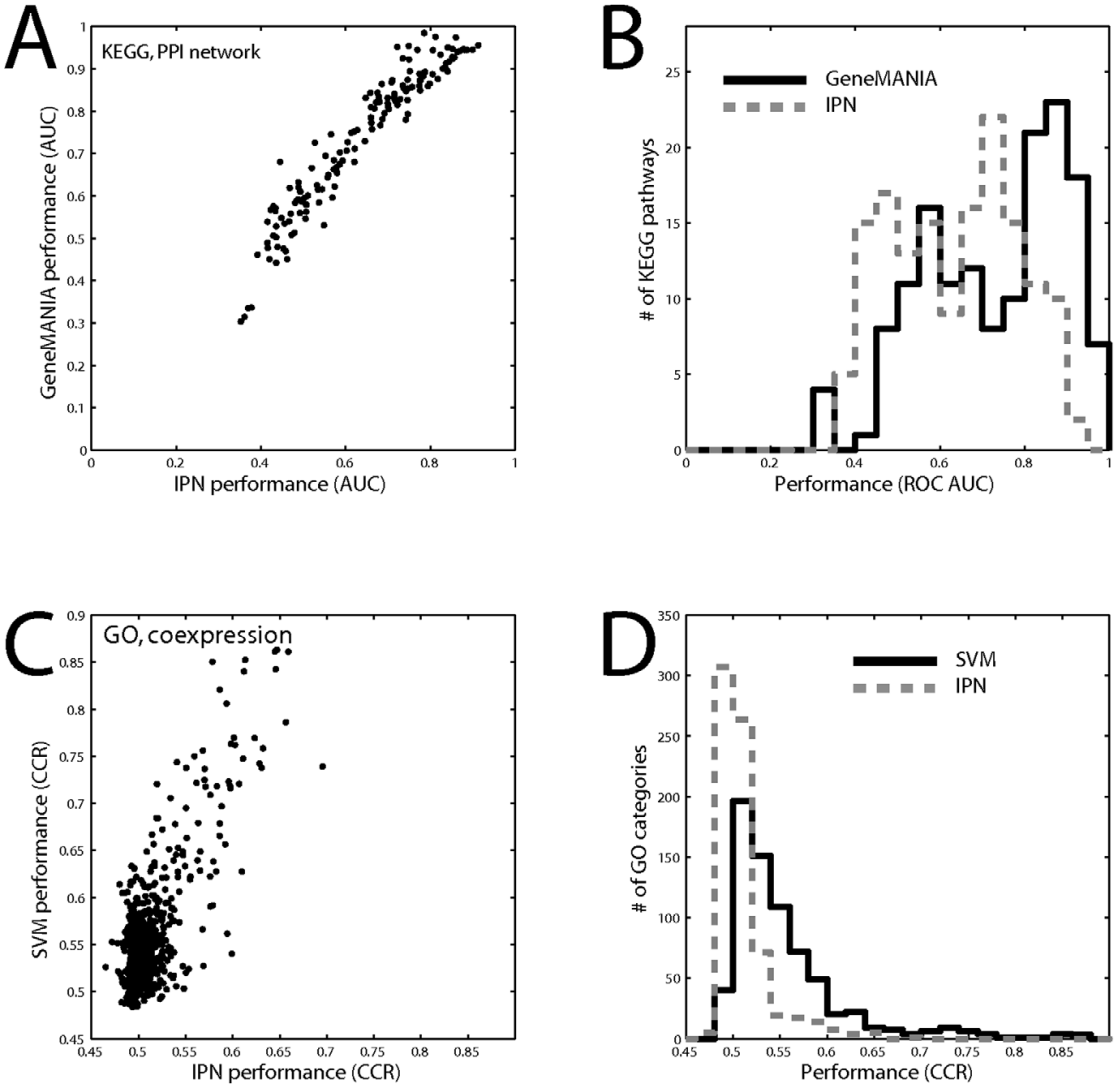
Individual property network (IPN) performance. Individual property networks are constructed by thresholding the self-outer product of the node degree for a given association matrix. A) The performance of an IPN protein interaction network is compared to the performance using GeneMANIA for the interaction network across all KEGG groups. B) The true protein interaction network performance is high but comparable to its IPN performance. C) The performance of an IPN using coexpression data across GO groups. D) The coexpression matrix gives low performance across GO groups but it is IPN performance is considerably worse. Only data in which some interaction is present within a given GO group – the learnable groups - is shown.

As with the simple node degree ranking, we found that IPNs perform very well in gene function prediction tasks compared to results from the original association matrix. Groups that perform well in the “real” analysis can be largely explained by variation in the performance of the IPN. Two examples are given in Figure 3. Prediction of KEGG pathways from protein interactions using GeneMANIA yields the highest average performances of all tests (Table 1), but only slightly better than the IPN (Figure 3A, B). In contrast, predicting GO from coexpression using the SVM (evaluated using corrected classification rate, CCR; see Methods) does substantially better than the IPN, but “real” performance is not as good and there are many groups which are not learnable from either network (Figure 3C, cluster of points around 0.5, Figure 3D). To confirm that these results are not influenced by the use of any specific algorithm, we performed a similar analysis using only the semantic similarity of gene pairs in the original data and the corresponding IPN to show that functional overlap in prediction is primarily due to multifunctionality bias (Figure S3).

**Figure 4:**
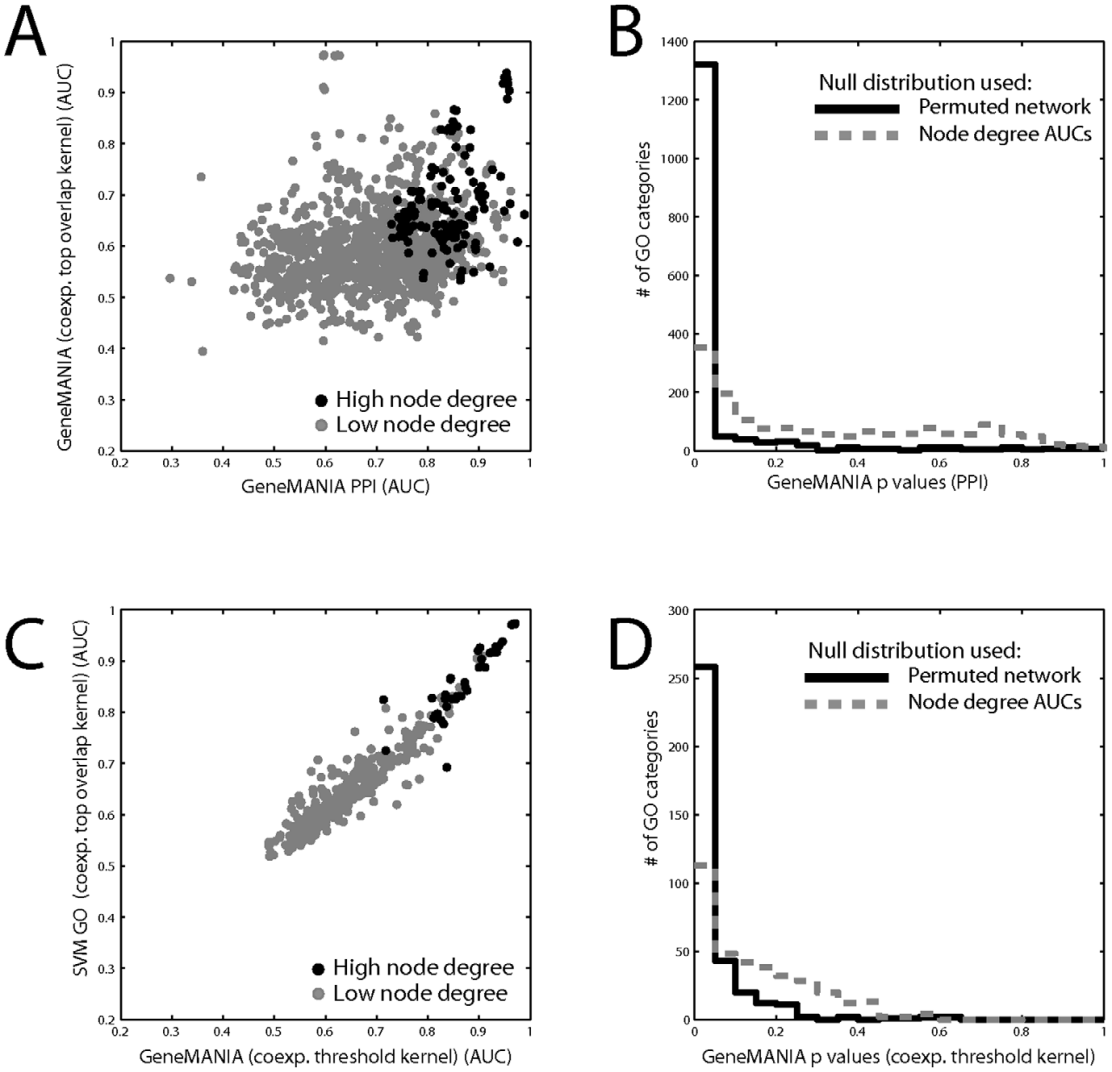
Controlling for node degree. A) True prediction performance is compared for the same method (GeneMANIA) using entirely different strongly predictive data sets (Protein interaction vs. coexpression) across GO groups. The performance is quite different, but when average node degree is higher in both datasets (top 5% by product, dark circles), then common performance is high. B) The statistical significance of the protein interaction results are computed using the usual permutation test (black) and using the distribution generated by the node degree scores (grey). C) True prediction performance is compared using the same underlying data (coexpression), processed differently (threshold vs. top overlap) and analyzed differently (SVM vs. GeneMANIA). Performance is strongly similar. D) The statistical significance of the weakly performing threshold coexpression results are computed using the permutation test (black) and the node degree vector (grey). Only data in which some interaction is present within a given GO group – the learnable groups – was used.

One prediction of the model we propose is that if performance is due to the node degree, varying the data will have a much more significant effect than varying the method, performance metric, or even the exact learning task. In Figure 4A, the same task is performed with two different high- performing association matrices, and there is little similarity in performance across all groups. However, the top performing groups are the ones in which the average node degree is high in both data sets (measured as the product of the average node degrees; black points in Figure 4A). In contrast, two different methods yield very similar results even on rather different tasks, so long as the same underlying data is used (Figure 4C), and again the best performance is for the groups with the highest node degree.

**Figure 5:**
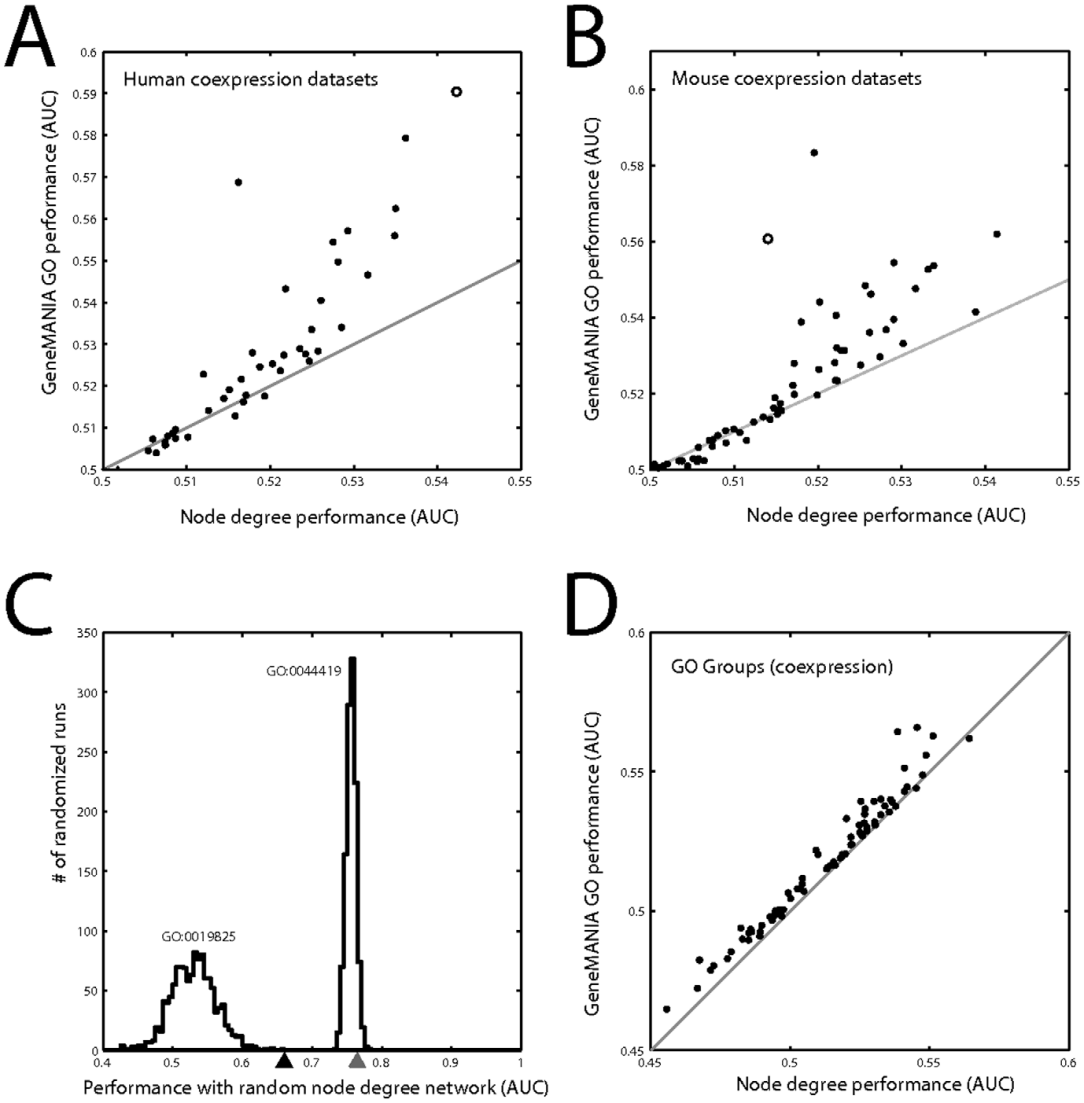
Performance across experiments. A) Coexpression performance in GO slim assessed as a function of using node degree ranking in human microarray datasets. Each point represents a single experimental set, with the open circle representing the aggregated association matrix. The identity line – representing no improvement beyond performance using node degree alone – is shown. B) Coexpression performance assessed as a function of node degree ranking in mouse microarray datasets. The open circle is the Su et al dataset. C) Random association matrices of fixed node degree were generated to generate a distribution of performances for two GO groups. Performance predicting membership using the original interaction matrix network is shown by arrowheads below the X axis (black: GO:001985; grey: GO:0044419). D) Performance of each GO slim group in the human data is plotted as a function of performance of the group using the node degree ranking.

To further address variability across data sets, we analyzed coexpression in the 47 microarray studies underlying our human gene coexpression network. We also examined 77 mouse microarray studies on the Affymetrix MOE430A platform, analyzing genes intersecting with the mouse Golden Path list (mm9) and the genes analyzed by Su et al. [60]. The Su dataset is of interest as it was used in a large evaluation of gene function prediction methods [40]. For computational efficiency, we only used the GO-slim categories in this analysis, which may also forestall concerns that our estimates of performance of node degree are enhanced by redundancy in GO (Figure S4). We found that overall, human dataset performance is strongly predicted by node degree (correlation of 0.94) with a variable fraction (approximately half to two-thirds) of the performance accountable by node degree performance (including in the meta-analysis dataset and protein-interaction dataset) (Figure 5A). A similar effect is present for mouse, with the Su dataset being an apparent outlier (Figure 5C).

Since node degree predicts gene function using multifunctionality without association information, its prediction performance could be used as the null distribution in computing p-values for the true association matrix, instead of using a permuted matrix as the null distribution. When we do this, it drastically reduces the number of significantly predictable GO groups in even the best of cases (Figure 4B and 4D). However, we have seen that different gene groups perform differently depending on the node degree of the genes within them (and that node degree of genes predicts performance). Thus, the null distribution must more properly be specific to the gene group, depending on the node degrees of the genes within the group. We developed a simple algorithm to generate random association matrices where the node degree for each gene is maintained (in contrast to usual permutation methods where the node degree distribution and network structure are maintained, but node degree for any given gene is allowed to change). Generating 1000 such matrices and testing prediction performance for the gene group on each gives a distribution of AUCs that is an estimate of performance under the null hypothesis of performance solely from multifunctionality effects. As can be seen in Figure 5C for two GO groups, some groups can have nearly all of their performance due to true guilt-by-association, though performance is not very good (GO:0019825, AUC = 0.67, p<0.001), whereas high-performing groups may simply have high average node degree (GO:0044419, AUC = 0.78, p∼0.035). Finally, in Figure 5D, we plotted average performance across all human datasets for each GO-slim category to show that each gene function prediction category has a unique degree of learnability from multifunctionality. Most groups lie slightly above the identity line, indicating some information gained from using the original network, but deviation from the line is low.

In this section we showed that much of the performance of guilt-by-association methods can be explained by multifunctionality. Data sets which perform well in gene function prediction are the ones most strongly influenced by multifunctionality. Isolating those predictions which are not explained by multifunctionality leaves a gloomier picture of the feasibility of gene function prediction from networks. In the next section we consider the generality of our findings.

### Assessing the generality of multifunctionality effects

A potential criticism of our results thus far is that we rely on the AUC or the CCR for evaluation. Focusing on the AUC, a common complaint is that the AUC takes into account true positives that are not near the top of the ranking, but low-ranking predictions are of no interest from a biological validation standpoint. Thus is it reasonable to ask whether the genes with the highest rankings in our predictions are multifunctional. Because the trend in which multifunctionality is predicted by node degree appears to be true not just for "hubs" but across all node degrees (Figure 1), we would expect that predictions based on node degree would still account for a significant fraction of performance when focusing on the top of the list.

We re-assessed node degree effects in the human protein interaction by three alternative methods. First, we employed the ROC50, a variant of the ROC that examines only the rankings up to the 50^th^ false positive example [61]. ROC50 performance averaged across GO groups was 0.64, while ROC50 using only node degree was 0.58 (significantly different from 0.5), confirming the prediction. ROC50 is not very widely used and emphasizes details of the order of the genes in the top of the rankings, which might not be of much interest. An alternative measure is positive predictive value (PPV), the fraction of true positives above a chosen threshold. To give a point of reference, using a threshold of 50 genes, GeneMANIA gave a mean PPV across GO groups of 3.8%, a 14-fold improvement over that expected by chance. We then generated a ranking of genes based on multifunctionality, using PPV instead of AUC as the optimization criterion (see Methods). This single ranking gives an average PPV of 4.7% averaged across GO groups. The final measure we examined is the area under the precision-recall curve (AUP). The multifunctionality-ranked gene list gives a mean AUP of 0.037, considerably lower than GeneMANIA's mean performance of 0.10. The difference between this result and the PPV result suggests that GeneMANIA tends to rank true positives higher than the multifunctionality ranking, though clearly both tend to rank positives more highly than expected by chance. However, this does not mean that using AUP gets around the problem of multifunctionality. Because AUP strongly rewards a single true positive at the top of the ranking, overall performance across GO categories is highly sensitive to the impact of a correct prediction for a highly multifunctional gene. To demonstrate this we constructed a gene network that contains only 100 edges (among 188 genes), with edges chosen based on the number of shared functions between the nodes. This yields a mean AUP of 0.07 (at 10 fold cross-validation). It turns out that 21 of these edges are in the real network. A closer examination of these results suggests that most of the GO categories that have high AUPs with GeneMANIA can be accounted for by the effects of just a handful of highly multifunctional genes. A full exposition of this effect is beyond the scope of this paper, but we also note that AUP suffers from having different expected values for each size of GO group and, unlike AUC, is also sensitive to the evaluation setup in sparse networks, improving at higher numbers of cross-validation splits (i.e., 10-fold vs. 3-fold), complicating its interpretation when attempting to detect trends. Altogether,these results strongly argue that the effects we report reflect a real tendency of multifunctional genes to account for predictions made, regardless of evaluation metric.

As a further test of generality, we examined another “gold standard” set of seven yeast gene interaction networks, some of which are aggregated from other (potentially overlapping) network studies data [22], [29], [30], [32], [62], [63], [64], [65]. Importantly, these are networks built using a variety of methods including genetic interactions, coexpression, protein interactions and other approaches. Cumulatively, these interaction networks and the data underlying them have been cited over 6000 times (based on Google Scholar). We used yet another measure of “performance”, the Dice-Jaccard semantic similarity (e.g., [66]) to directly assess the quality of the network connections without performing machine learning. The links within the real networks are highly significant individually or in aggregate, possessing much higher semantic similarity than random links (Table 3). However, as predicted by our previous results, a large fraction of this performance may be explained purely by multifunctionality biases. This is true even in the case of YeastNet [65] which, unlike the other networks we tested, was tuned using information from GO to improve functional relevance. The aggregate network node degree predicts all Gene Ontology categories in yeast with an average ROC of 0.63, comparable to what we observe with the human data. Moreover, the aggregate network IPN accounts for roughly three-quarters of the performance of the true yeast network connections (61% higher in the IPN than random). In the individual networks (e.g.,Figure S5), the distribution of scores for individual links can be seen to be strongly shifted to higher values both for the real and IPN networks; using the IPN to estimate false discovery rates would substantially increase false discovery estimates. There is no gold standard method to assess network performance, but the Dice-Jaccard index attempts to correct for prevalence/multifunctionality (it is normalized to number of terms), and thus it tends to reduce IPN contribution over other methods, such as GO term overlap. These results show that the structure of these networks strongly reflects multifunctionality, and that gene function predictions stemming from them are heavily influenced by this fact. They furthermore show that our findings extend to all networks we have examined, and are again not merely a function of assessing the networks using machine learning and predictive performance as outcome measures.

**Table 3:** Yeast network performance.

	Yeastnet	MPACT	DIP	MINT	BIOGRID	Costanzo	Fields	Aggregate
True network	0.46	0.42	0.40	0.45	0.65	0.30	0.43	0.41
IPN	0.41	0.32	0.31	0.33	0.40	0.29	0.31	0.37

Table 3: Seven yeast networks were assessed according to the Dice-Jaccard coefficient of the links they select. All exhibit strongly functionally significant links compared to random data which possesses a Dice-Jaccard coefficient of 0.23. However, a large fraction of this performance is present in the individual property networks (IPN) which also exhibit strongly significant links. The network from aggregating the true networks and IPN networks is shown. Aggregation is accomplished by including all links present in the original networks; thus, the aggregate of the true networks includes all network links present in the true networks.

### Can the impact of multifunctionality be reduced?

Node degree has been previously recognized as potentially influencing functional predictions. Indeed, it is important to note that the first step in GeneMANIA's operation is to attempt to correct for node degree (downweighting each edge by node degree). As discussed in the introduction, a variety of methods have been suggested to minimize the importance of node degree. Above (i.e., Figure 4), we've considered one of the most general approaches, which is constraining node degree to take a much more restricted range of values by top overlap. However, this has little effect because it is the AUC produced by the node degree rank that matters (which reflects the influence of multifunctionality), and top overlap tends to preserve node degree ranks (r>0.76 in coexpression). Here we consider other possible corrections for node degree biases and show that their interpretation is still enhanced by consideration of the effects of multifunctionality.

We first considered the role of sparsification. Sparsification is a necessary step for methods like GeneMANIA, and protein interaction networks are inherently sparse, but coexpression networks begin as a complete distance matrix. We hypothesized that that sparsification induces network structures that affects node degree/multifunctionality issues. In particular, sparsification is likely to result in high node degree for genes with higher variability (even with similar means). In un-sparsified data, variation in mean interactions across many genes may dominate over the heaviness of the tails. Therefore we assessed the impact of multifunctionality on non-sparsified coexpression data. We used a simple nearest-neighbor gene function prediction algorithm which could work rapidly with unsparse data.

**Figure 6:**
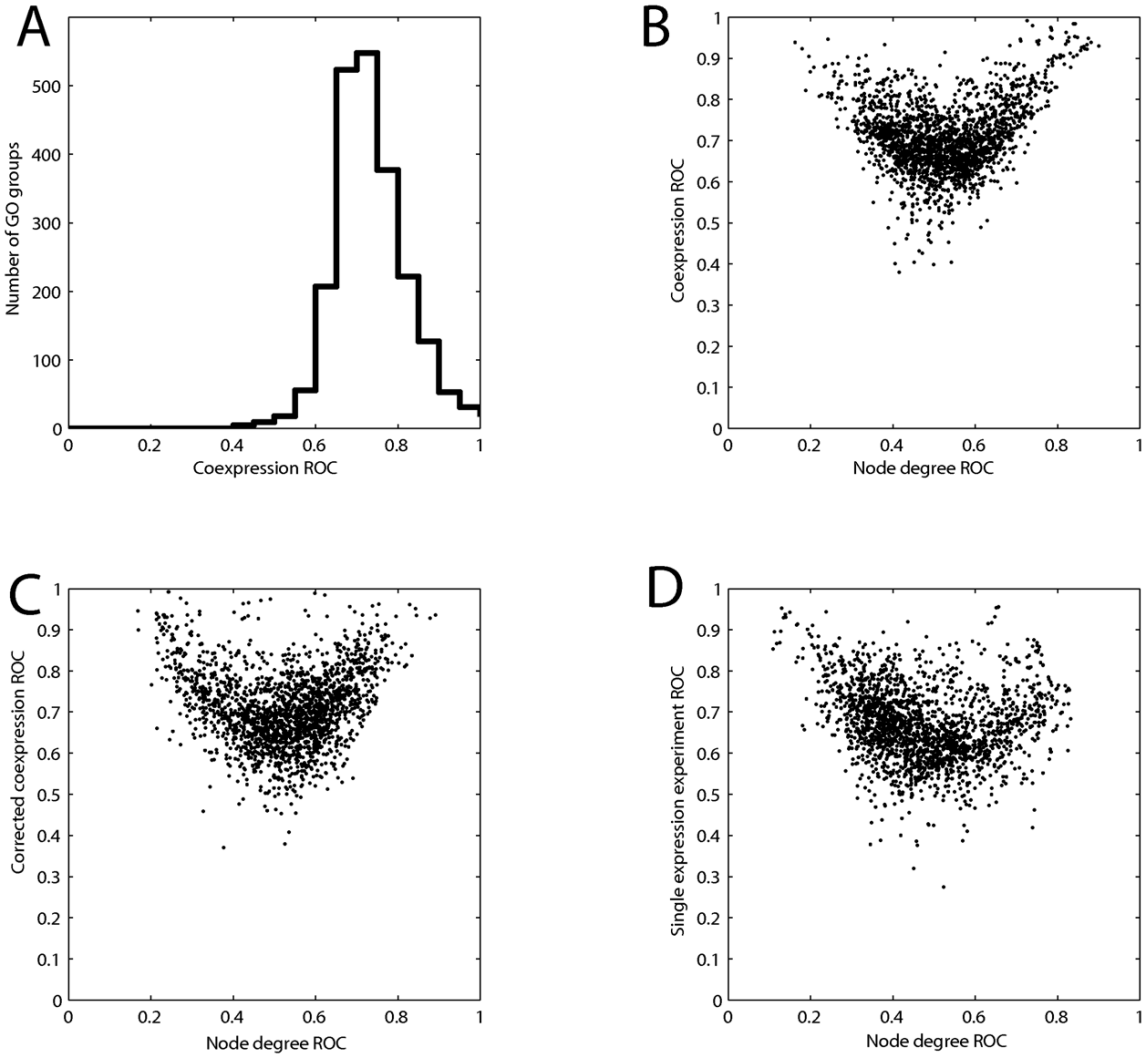
Potential node degree corrections do not remove node degree influence. A) The unsparse coexpression data exhibits low node degree bias and very good performance using a nearest neighbor voting algorithm (mean AUC of 0.71). B) A very strong dependency on node degree is retained in the nearest neighbor analysis. The more extreme the performance of a GO group is based on node degree, the better the performance in the nearest-neighbor analysis. C) After a correction to remove the influence of node degree – links included only if performance is above that predicted by node degree alone – and aggregation, this matrix exhibits high performance (mean AUC = 0.70) and modest node degree bias (mean AUC = 0.55), but it again exhibits the triangular dependence on multifunctionality. D) An unsparsified coexpression network from a large multi-tissue coexpression experiment (GSE7307) yields a mean AUC of 0.68 across all GO groups and no node degree bias in favor of multifunctionality (mean AUC = 0.48), but exhibits the same triangular distribution of node degree AUCs.

The unsparse coexpression data exhibits low node degree bias (the mean AUC based on node degree ranking is 0.52) but very good performance using the prediction algorithm (mean AUC of 0.71, Figure 6A). Thus this method might appear to remove the influence of node degree, and thus multifunctionality. However, there is still a very strong dependence on node degree (Figure 6B). The more extreme the performance of a GO group is based on node degree, the better the performance in the nearest-neighbor analysis. Thus we can see that GO groups are predicted if their multifunctionality is either very high or very low (that is, the genes in the group are largely mono-functional).

In Figure 6C we show results for another correction for node degree, using sparse data made up by aggregating multiple coexpression networks. Each coexpression matrix in the aggregated coexpression matrix was first normalized so that edges are retained only if the nodes exhibit a correlation above that predicted by their node degree alone (e.g., as in [67]). After aggregation, this matrix exhibits high performance (mean AUC = 0.70) and modest node degree bias (mean AUC = 0.55). However, it again exhibits the triangular dependence on multifunctionality – highly predictable groups tend to either contain unusually multifunctional genes or unusually mono-functional genes.

As a final test for the influence of node degree, we examined in more detail the highest performing individual data set (excluding protein interaction aggregate data) across all methods and data-types. This was an unsparsified coexpression network from a large multi-tissue coexpression experiment (GSE7307). It yields a mean AUC of 0.68 across all GO groups and no node degree bias in favor of multifunctionality (mean AUC = 0.48). However, even in this case, the same triangular distribution of node degree AUCs is apparent (Figure 6D). Thus, the set of high performing functional groups generally consist of two quite different classes, both understandable with the consideration of multifunctionality.

Taken together, the results in this section demonstrate that attempt to reduce the effect of node degree bias do not have the desired effect, nor are data sets which appear to have no node degree bias actually free of its effects.

## Discussion

Multifunctionality has arisen previously in discussions of gene function as both an important biological principle (i.e., pleiotropy) and central factor in network structure (i.e., hubs), and as a potential source of spurious results (i.e., promiscuous proteins). In this work we have suggested that most functional assignment is driven by multifunctionality or must be considered in the context of the degree of multifunctionality.

### Multifunctionality and promiscuity

Our results strongly suggest that there is a general problem in the interpretation of guilt-by-association analyses. The effects of multifunctionality trickle down into association networks resulting in learnability that has nothing to do with the specifics associations. Even at very stringent ROC thresholds, just because a set is learnable, we can not be confident that we have gained information regarding which genes are specifically associated with the set, and new predictions will mostly be based on the effect of multifunctionality. This does not mean the predictions are necessarily biologically wrong, and on the positive side, the gene learning algorithms can still do better than the node degree list, meaning they extract useful association information. However, where possible the results of the analysis should be interpreted with reference to the node degree property, not the network property, essentially because we should disfavor a complex model when a simpler model will suffice [68]. Learning from the IPN or the node degree ranking is like relief of symptoms from a placebo – significant and interesting, but potentially misleading with respect to causality. We stress that our results do not depend on whether one believes that multifunctionality is a true property of genes reflected in the patterns of annotation, or some kind of artifact of how genes are studied and annotated. The question of what is the “true” multifunctionality of genes separated from possible biases in patterns of research or annotation is a topic for future research.

### Multifunctionality as hubs

The practical importance of the bias is illustrated by our ability to predict associations of genes with genetic conditions from OMIM using nothing but the number of Gene Ontology terms a gene has. Because we take the OMIM set to be less likely to suffer annotation biases than GO, the strength of this effect comes closer to reflecting the degree to which multifunctionality is a necessary biological consideration. The causes of this effect are likely to be complex and require further study to understand completely. For some genes, their presence on candidate lists might be influenced by biases in interpretation of genetic mapping studies, in which well-known genes are chosen for follow-up genotyping. For genes which have unequivocal roles in disease, they might become well studied and thus accumulate more GO terms. On the other hand, biases could take the form of false negatives – there may be additional genes which are not yet implicated in a given disease because they are difficult to study. Finally, genes which are important for disease processes might in fact be “hubs” (a notion with support in the literature [69], [70]) and tend to be important for multiple diseases. Whatever the source of bias, it poses a problem for tasks like “gene prioritization” in genome-wide association studies, for which there are a growing number of algorithms and tools. Our results suggest that without correction for the prevalence bias induced by multifunctionality, these methods will tend to prioritize genes which have the best existing characterization, and be insufficiently sensitive to the choice of target disease. Indeed we expect that all gene function prediction methods that rely on some form of “guilt-by-association” will have similar difficulties. The implications also reach beyond analyses that use networks. A preliminary analysis of differential expression studies (not shown) suggests that the probability that a gene is identified as differentially expressed is in part accounted for by multifunctionality. This further suggests that the popular approach of gene set enrichment analysis [71] will also tend to produce rankings affected by the degree of multifunctionality.

### Correcting for multifunctionality

Could this source of bias be removed? As we observed, the problem is worse for some data sets than others (Figure 5A and B, Table 3), but the underlying reason for this is not yet clear. It is possible that alterations to study designs or data preprocessing might improve matters. However, it is unlikely that attempts to filter the final network itself will be fruitful. The appearance of the bias generated by multifunctionality could be completely removed by artificially forcing all genes to have the same node degree. While this successfully shoves the problem under the rug, it will almost certain degrade predictive performance and destroy any real information there is in variability in the number of associations a gene has. Less drastic schemes might result in less loss of information, but will inevitably be less effective at removing the bias. Due to the current difficulty of simply correcting for multifunctionality, our focus has been on how to control for it.

Using the node degree vector or IPN to determine an average performance across many gene groups is useful for validating gene function prediction algorithm performance, but how would a biologist using a prediction method for a specific function evaluate the results? In this case, we recommend comparing the ranking the algorithm gave them to the optimal gene ranking from GO (Figure 1A). If there is similarity between the rankings, the algorithm-derived predictions cannot be assumed to be meaningful. In general our results will hold over any set of genes exhibiting heterogeneity with respect to prevalence and will be much stronger where prevalence varies more (e.g., if we retain hypothetical proteins in our analysis, it strongly increases control performance).

While we focused on problematic aspects of prevalence biases, the most multifunctional genes are clearly worthy of study. Although we have recommended rejecting them from gene function results as unlikely to be *specifically* relevant to the question at hand, that is only because they may play an role in a many contexts/pathways (e.g., hub proteins [18]).

To assist others in addressing the issues we raise, we have provided the rankings of genes by multifunctionality and other information including Matlab™ implementations of the algorithms as Supplementary Information and at http://www.chibi.ubc.ca/Prevalence.

## Methods

Evaluation of prediction performance: We used the AUC ROC as our main measure of performance in prediction. An AUC of 0.5 represents classification at chance levels while and AUC of 1.0 is obtained for a perfect classifier. In the gene function prediction literature, values >0.7 are considered good and values >0.9 are atypical. Additional measurements considered included ROC50 [61], correct classification rate (CCR), positive predictive value (PPV), and area under the precision-recall curve (AUP). The top 100 network for AUP evaluation was constructed by adding connections between genes with many overlapping GO groups. For each connection added, GO groups were weighted by the inverse of the number of times they had already been used in the network.

Gene lists: We analyzed the list of human or mouse genes from the UCSC GoldenPath database "known gene" table intersected with the microarray platforms used (independently in human and mouse analysis). Thus, we analyzed 15439 of the 18534 known human genes and 12513 of the 18592 known mouse genes. Alternatives such using the full microarray gene list or full known gene set generally yielded even stronger prevalence biases (stronger prevalence list performance and stronger correlations between prevalence and true performance). The 6200 known yeast genes were obtained from NCBI.

Gene Sets: Human Gene Ontology annotations consisted of 10127 gene sets with 1838 sets having between 20 and 1000 genes within them; following Figure S6, it was this subset used in analysis. All analysis used only the GO groups with 20–1000 genes, except where specified otherwise (e.g., Data S1 Section 4). 558 genes were downloaded from the Alzheimer's database, ALZgene database [55]; 769 genes from the Schizophrenia database, SZgene database [56]; and 191 genes from the autism database, AutDB [58]. The 217 KEGG human gene sets were obtained from the KEGG webservices [72]. The complete OMIM human disease table was downloaded with 4069 diseases having genes in the known gene table [59]. The GO slim set was obtained from the GO website, consisting of 127 GO categories [28]. Performance with GO slim was confirmed to be nearly identical to full GO performance.

Gene Prediction Algorithms: GeneMANIA was used without a negative training set across each training gene set with three-fold cross-validation to determine a ranked list scoring genes as to how well they belonged within the known gene set. Eight-fold and n-fold cross-validation was also performed on the GO categories using the top-overlap coexpression data to confirm that higher fold number did not alter results. For support vector machine (SVM) classification, the default Matlab implementation was used with default settings. For the SVM, the correct classification rate (CCR) was used as the performance metric. Due to computational constraints, SVM was implemented across the complete gene set piecewise, in sets the same size as the training set, so that the CCR almost parallels the ROC AUC in meaning. Thus, a set of 100 genes would have added to it 100 random non-set genes and SVM performed on the association matrix among these genes and CCR measured using a full hold-out validation. This was performed using multiple sets of non-set genes until all genes were tested, and the CCR averaged across runs. This produces more confident estimates (heavily averaged), but also reduces the information available to the algorithm in making each classification. For nearest neighbor analysis, an implementation was written in matlab which ranked genes by a voting scheme within the training set (by ranked coexpression) relative to genes outside the training set. The sum of coexpression ranks between the training set and the candidate gene was divided by the sum of coexpression ranks between the genes outside the training set and the candidate gene to determine degree of candidacy.

Semantic Similarity: Semantic Similarity was assessed using Gene Ontology term overlap (in Data S1) and by the Dice-Jaccard index (Gene Ontology overlap normalized by the number of terms attached to gene A plus the number of terms in gene B). Methods of semantic similarity are broadly correlated [66], and Dice-Jaccard index tends to be conservative with respect to estimating prevalence bias; that is, underestimating its effect. If one wished to be conservative in reporting novel results with respect to biases induced by multifunctionality, the less stringent GO overlap measure may be more appropriate.

Gene networks: Our human protein-protein interaction network was obtained from InnateDB [73] and contained 74932 interactions among 9180 genes. Individual yeast networks were downloaded from their respective websites [22], [29], [30], [32], [62], [63], [64], [65]. In each case, the network was treated as a set of interactions across the gene set used; however, intersecting again to only genes with interactions in the network left the proportional effect of prevalence bias approximately the same. Our coexpression matrices were obtained from publicly available microarray expression experiments analyzed in a microarray meta-analysis system (Gemma, http://www.chibi.ubc.ca/Gemma). In coexpression networks, correlation values are typically thresholded to select the edges that make up the final network; this is the “thresholded” matrix. A commonly-used alternative is to choose only overlapping nearest neighbors; we call this the “top overlap” matrix. Available as Data S1 are the list of 232 individual human experiments used to construct the full Gemma matrix, the 47 GPL570 experiments used to construct the thresholded and top-overlap matrices, and the 78 mouse experiments used for individual analysis. Briefly, Gemma uses a threshold of 0.5% on the correlation between expression profiles of genes to determine coexpression, or a p-value from the Fisher transformed correlations, whichever is more stringent [34]. The full Gemma matrix is the sum of the individual coexpression matrices. In keeping with the Gemma threshold and close to that used in the GeneMANIA paper [39], our default sparsity was 0.5% when combining the 47 GPL570 experiments. In the threshold association matrix, this is obtained by using a fixed number of experiments coexpression must be present in (the sum across experiments). In the top-overlap association matrix, a threshold is chosen for each gene to provide 0.5% sparsity for it, and then only common associations included. Alternatively, one could choose a per-gene sparsity that when required to be top-overlap, matched the overall 0.5% sparsity; however, variations in sparsity were not a significant factor in our results in this range. The data displayed in the figures were filtered to remove function categories in which there was no coexpression within the group (to remove artifacts such as a line of points at 0.5 using SVM for which there were no significant predictions) – however, all numerical values given in the text and the tables use the full set of results. This filtering typically altered results by in the range of 0.01 (e.g., correlation between node degrees and true results changed from 0.95 to 0.96 when filtered or AUC went from 0.59 to 0.60). Only in the case of threshold coexpression performance was there a substantial shift in performance (from 0.55 to an AUC of 0.67).

Individual Property Networks: Given an association matrix, the fixed ranking was constructed by summing across one dimension of the association matrix and ordering the genes by this associated node degree. The IPN was constructed by taking the self-outer-product of the vector sum across an association matrix representing node degree. A fixed threshold that generates the same sparsity as the original (after the addition of identity relationships) was then applied. Note that even for an association matrix generated by top-overlap, its IPN should be generated by threshold (the default assumption, as in Data S1, Section 2, is that the scores provide rank across the entire matrix). Our Supplementary Methods cover the construction of random matrices of matching node degree as well as other details.

## Supporting Information

Data S1 **Sections 1-7. **Section 1: Construction of the optimal single gene ranking; Section; 2: Construction of the Individual Property Network (IPN) Section 3: Effect of microarray platform gene representation on coexpression; Section 4: Effect of GO group size and network sparsity; Section 5: Mean, variance, and statistical significance of AUCs; Section 6: Absolute Performance; Section 7: Supplementary Methods
(DOC)

Figure S1 **Predicting OMIM condition using the optimal gene ranking derived from GO.** The distribution of AUC values is shown when predicted using the same optimal vector used in Figure 1A.
(TIF)

Figure S2 **Schematic of construction of the Individual property network (IPN).** Top: an original sparsified association matrix (black  =  association). Middle: Outer product of the Associability vector. Bottom: After processing the outer product to yield an “association matrix” of equivalent sparsity to the original data.
(TIF)

Figure S3 **Semantic similarity.** Semantic similarity allows us to assess common gene function in an association matrix without the use of a prediction algorithm. Using common GO term overlap, IPN performance is superior to original network performance.
(TIF)

Figure S4 **Performance of the optimal list is insensitive to using subsets of genes.** Genes were randomly selected and the optimal list for those genes was generated. Top: The performance of the optimal list is strong even for very small groups of genes, with standard deviation shown by the grey region. Bottom: The error generated by using the optimal list over all genes is shown as a function of the number of genes included in analysis. Even for small groups, using the original optimal list is a reasonable approximation of constructing a specific list representing the subset of genes used.
(TIF)

Figure S5 **Performance as a function of number of genes.** Gene function performance is plotted along with standard deviation with increasing GO size.
(TIF)

Figure S6 **Yeastnet distribution of semantic similarities.** The semantic similarity distribution over all links in the dataset is shown, as well as the similar distributions for random data and the Individual Property Network.
(TIF)
