# Supplementary material for: The Impact of Multifunctional Genes on "Guilt by Association" Analysis
Source: PLoS One. 2011 Feb 18;6(2):e17258. doi: 10.1371/journal.pone.0017258 (PMC3041792; doi:10.1371/journal.pone.0017258)
Supplement: Data S1 — Sections 1-7. Section 1: Construction of the optimal single gene ranking; Section; 2: Construction of the Individual Property Network (IPN) Section 3: Effect of microarray platform gene representation on coexpression; Section 4: Effect of GO group size and network sparsity; Section 5: Mean, variance, and statistical significance of AUCs; Section 6: Absolute Performance; Section 7: Supplementary Methods (DOC) [file pone.0017258.s001.doc]

# Supplement to Gillis and Pavlidis

# Section 1: Construction of the optimal single gene ranking

Here we provide an explanation of how the optimal gene ranking for predicting GO groups was obtained, along with an informal proof that it is indeed optimal.

Our measure of performance is the area under the ROC curve (AUC). The formula for the AUC for a GO group given a ranking of all genes can be written as the two-sample Mann-Whitney U test, that is:


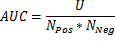


Where N_Pos_ is the number of genes in the GO group, and N_Neg_ is the number of negatives. Using the formula for U, taking rank index 1 to be best across *n* genes, we have for GO group j:


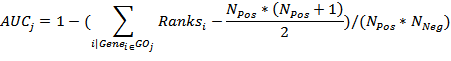


We wish to maximize this, summing over all m GO groups. We have:


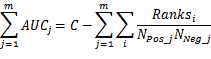


Where C simply collects the constant terms and NPos_j is the number of genes in GO group j. The sum can be conveniently rewritten as a product of a weighting term for each GO group, the binary classification matrix of Genes by GO groups (with 1 representing presence of the gene in the GO group), and a rank vector representing the rank of the gene in the list:


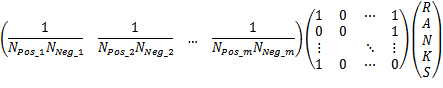


We wish this formula to take the minimum possible value. Call the product of the first two terms the “score vector” of values for each of the *n* genes given by:


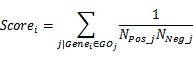


The mean AUC across all GO terms is thus maximized when the inner product of the rank vector and the score vector is minimized. This occurs when the ranks run opposite to the scores (following). If the weights are ignored, this gives a ranking by the number of GO terms; as suggested by the derivation, empirically including the weighting term yields a better ranking.

Now we show that ranks run opposite to scores. By contradiction, if that rank does not minimize the product, then some permutation of ranks exists which produces a lower inner product. Since every permutation can be represented as a product of transpositions, this implies at least one transposition would product a smaller inner product. That is:


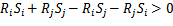


Where R_i_ is the pre-transposition rank at position i and R_j_ is the pre-transposition rank at some later position j, and similarly for the score vector S. We factor to obtain:


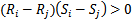


But if the scores are ordered from highest to lowest, the ranks are ordered oppositely, so that if R_i_>R_j_ then S_i_<S_j_ we have a contradiction. So, ranking the genes by the score vector (from highest to lowest) will optimize the mean AUC.

This is intuitively consistent with our multifunctionality model. First, the score is very well approximated by simply ignoring the weighting term and taking the unweighted sum (i.e., the number of GO terms for the gene, the simplest notion of multifunctionality within GO). However, it also makes sense that even in a model in which genes have a base probability for appearing, that probability should be weighted by the number of genes that appear in the test in some ways. That is, the probabilities of the genes appearing in the test could be sensibly weighted by the number of genes that appear in the test altogether (without involving function in any way since all genes would be equally weighted). Likewise, being the one gene not to appear in a test is more informative of relative multifunctionality than in being one of many such genes (as also follows from the symmetry of ROC AUC). Thus, the weighting term makes intuitive sense as well.

### Section 2: Construction of the Individual Property Network (IPN)

In this work we propose that if gene function can be learned using guilt-by-association from a non-network property of genes, then the effect of guilt-by-association in the original network must be discounted. In particular as we propose a simple model of “multifunctionality” as the relevant non-network property, with node degree as an estimate of the multifunctionality. To test the relevance of this property to learning from networks, a new matrix is reconstructed as the outer product of normalized node degrees (the mean across columns of an association matrix, or node degree over number of nodes) with itself to generate a matrix consistent with the multifunctionality model. We call this an individual property network because while it is a gene network, it is based entirely on an individual property of each gene. Here we explain in more detail how IPNs are constructed and an additional justification for using node degree to construct one.

Consider that each of n genes g_i_, i ∈ {1…n} in a coexpression or protein interaction adjacency matrix C can be characterized by a single number. We characterize the list of genes by an individual property vector *b*  - the ordered list of numbers associated with each gene. The outer product of this vector with itself (*bb^T^*) generates a new n x n matrix B in which the value B_ij_ is high when both genes *i* and *j* have high values, low when they both have low values, and intermediate if only one of them has as high associated value, etc. This new matrix can be preprocessed like the original coexpression matrix (e.g., thresholded) to form an IPN adjacency matrix that can be analyzed in the same way as the original data. Supplementary Figure 2 shows a schematic transformation from coexpression matrix to *bb^T^* matrix using node degree for b.

As before, consider the matrix in which each element is assigned a value p_i_p_j_ (a self outer product), reflecting only the independent probabilities of the genes being present. The vector that most closely matches the original network (up to a constant factor) uses the node degree vector. In other words, there is no other single vector *b* that provides a closer approximation to the original network under the “random interactions” model. This means that one could derive the motivation for using “node degree” entirely from the concept of finding the optimal *b*.

Following this line of thought, we wish to find *b* to minimize D in the following equation:


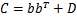


We take the error term to be sum of squares of the elements of D. Then we are seeking to find the vector *b* of length n to minimize the expression:


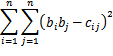


Where c_ij_ are the elements of the original association matrix. Setting the partial derivatives equal to zero gives us, for each b_i_:


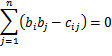


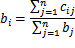


From this we see that the optimal *b* is equivalent (up to a constant normalization factor) to the node degree. Summing across all b_i_, the normalization factor is:


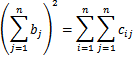


Thus, node degree is not only an intuitive individual property to use, but mathematically sensible. More importantly, it provides an alternative way to see node degree as an interesting underlying property of genes in networks.

### Section 3: Effect of microarray platform gene representation on coexpression

As mentioned in the paper (Figure 1C), node degree across all 232 human experiments in Gemma provides learnable GO information. We suspected that some of this effect was due to variation in representation of genes across different platforms: some genes are represented on many platforms while others are not, giving the latter fewer chances to demonstrate coexpression. To test this possibility we constructed an association matrix which purely reflected the joint presence of pairs of genes in a probe set (subject to the usual requirements for coexpression, e.g., not the same probes). This association matrix just measures the maximum number of times a pair of genes could be observed to be coexpressed in Gemma. This association yielded an average AUC of 0.54, which while modest is significant (p<0.01). Our interpretation is that genes which are more often examined in microarrays tend to be more characterized in GO and therefore susceptible to the bias documented in Figure 1A. Because of this effect, we limited our baseline coexpression meta-analysis to 47 data sets that use the Affymetrix Human HG-U133 Plus 2 Gene Chip (GEO accession GPL570), which covers most known human genes (the list of data sets used is available as supplementary data). This removes the effect of platform variation as a significant predictor of gene interaction and decreases the correlation between node degree and number of GO terms to 0.15. Another method for removing platform variation would be to examine each microarray dataset individually rather than in the aggregate, and we also perform this analysis in humans as well as mouse data (Figure 5A and B).

# Section 4: Effect of GO group size and network sparsity

Guilt by association requires the training set of genes to be functionally labelled, and might be reasonably expected to vary in performance subject to the amount of such information available. That is, it may be harder to learn a particular function for a gene if few genes with that function are known (and function is only defined by which genes are associated with it). Thus, it is usual to limit GO group analysis to GO groups of a certain size (e.g., ^1^). We examined the effect of GO group size in our baseline coexpression network. Surprisingly, mean performance does not improve substantially or progressively from its initial (2 genes) value as the number of genes in the GO group increases; however, the variability of performance declines progressively as the GO group size increases from 2 to approximately 20 genes per GO group. Based on this, we report subsequent results using only those GO groups containing 20 or more genes. We also excluded GO groups of larger than 1000 genes in order to capture reasonably specific gene properties; however, this criterion had no noticeable effect on any subsequent results. We also varied sparsity in all subsequent analyses (between 0.5% to 5% in increments of 0.5%). This had a minimal effect in reported results. In all subsequent analyses we use a sparsity of 0.5% as this is both close to that used in the sources for our data or methods (e.g., Gemma Database, ^2^), provided the best algorithmic performance among those tested values, and considerably speeded computation (by virtue of highest sparsity).

### Section 5: Mean, variance, and statistical significance of AUCs

To assess whether a group of genes is significantly learnable a null distribution is necessary. Rather than purely randomizing network structure, the standard protocol is to retain network structure and randomize gene/node identity; that is, a permutation test across genes (e.g., ^3, 4^). In the case of our default coexpression network used to generate ROC curves using GeneMANIA across GO groups, this gives a mean of 0.5 and an average standard deviation of 0.048 in the distribution of AUCs, in contrast to a purely randomized matrix of equivalent sparsity with average standard deviation of approximately 0.01. Of course, averaging across multiple runs reduces the standard deviation. The maximum value in the first run was 0.68, indicating that a conventional analysis would normally conclude any group of genes learnable above an ROC AUC of 0.68 to be extremely significantly learnable.

### Section 6: Absolute Performance

The absolute performance of GeneMANIA using the InnateDB protein interaction network was quite high (Table 1, Figure 2A), particularly considering that many groups were known a priori to be largely unlearnable due to the sparsity of data. In contrast to this, the performance of the aggregate thresholded coexpression matrix was quite poor (Table 2, Figure 3D). It might be tempting to conclude that protein interaction provides superior performance to threshold coexpression. However, our results do not support that interpretation, because very high proportion of the performance in the protein interaction network was attributable to node degree, while it is lower for the coexpression data.

In general, we find it is easy to modify methods in sensible seeming ways which improved gene function prediction performance, but all such modifications concomitantly increased multifunctionality bias. For example, including a larger number of different array designs improved performance, as did removing sparsely coexpressed (or interacting) GO groups – choices which may seem reasonable a priori. However, these improvements in performance (typically yielding an ROC of ~0.8) caused a similar increase in node degree performance (increasing the proportion of performance due to multifunctionality bias). Thus our choices to use a single array platform for the coexpression data tends to decrease multifunctionality bias, but also resulted in lower overall performance. In other words, by making different choices in the setup of the experiment, we could reduce the apparent performance gap between the PPIN and coexpression. But this is a placebo improvement. Not all improvements in prediction performance are equal.

Given the strong trend lines in Figure 5, the degree of performance attributable to multifunctionality is better thought of as a fraction than an absolute number. That is, our performance of 0.63 using the node degree of a protein interaction network does not indicate that any performance beyond this level in future experiments with other data is better than multifunctionality performance. It is potentially easy to improve performance just by increasing the placebo/multifunctionality effect.

### Section 7: Supplementary Methods

Matching node degree matrices were constructed by randomly assigning ones to an initial identity matrix till the node degree for a given position was filled. That is, initially a pair of random numbers are generated, drawn uniformly from the positions with node degrees greater than ones (self-connection). This position is then changed from zero to one (as is the value at the transpose position). Now, a pair of random numbers are again generated from the positions in which the node degree does not yet match the original data. The process is repeated till no further connections can be added. In practise, this will result in a slightly less than perfect match of the node degrees to the original matrix because the last few connections may already be ones. However, this effect is very minor. The matching node degree matrix is generally less suitable than the IPN or the single node degree vector for use as a control (since overly random).

We used SVM as a binary classifier, but node degree provides a ranked list of genes. In our use of GeneMANIA, this ranked list of genes can be directly compared to the ranked list of genes provided by the algorithm. Thus, our prediction is identical regardless of training data. This is not precisely true in our implementation of SVM. We used SVM as a binary classification scheme, but node degree does not provide a hard threshold. So, the hard threshold for the node degree uses the proportion of positives in the (unstratified) training data. Note that no details of the actual data in the training data or associations (which gene is associated with which, etc) are used, and the node degree correct classification uses the same ranked list regardless of training data.

GeneMANIA offers the option of including known, negative and positive genes in the training set. The choice of a specific negative set may influence performance but we treated all unknown genes equivalently (e.g., all labeled negative/unknown). Then we cross-validate by adding positive genes (piecewise) to the set of unknown and comparing the relative ranks. This may be thought of as asking how well an algorithm could reclassify existing genes. Our performance estimates should be slightly conservative (since actively misclassifying genes). The default GeneMANIA cross-validation retains known, unknown and negative in the training data but then ignores unknown for the purposes of cross-validation. Using the defaults slightly, but consistently decreases the absolute performance and increases the proportion of performance which may be attributable to multifunctionality (judging by AUC histograms).

References

1. Wolfe, C.J., Kohane, I.S. & Butte, A.J. Systematic survey reveals general applicability of "guilt-by-association" within gene coexpression networks. *BMC Bioinformatics* **6**, 227 (2005).

2. Mostafavi, S., Ray, D., Warde-Farley, D., Grouios, C. & Morris, Q. GeneMANIA: a real-time multiple association network integration algorithm for predicting gene function. *Genome Biol* **9 Suppl 1**, S4 (2008).

3. Lee, H.K., Hsu, A.K., Sajdak, J., Qin, J. & Pavlidis, P. Coexpression analysis of human genes across many microarray data sets. *Genome Res* **14**, 1085-1094 (2004).

4. Dobrin, R. et al. Multi-tissue coexpression networks reveal unexpected subnetworks associated with disease. *Genome Biol* **10**, R55 (2009).
