# Supplementary figures and images for: The Impact of Multifunctional Genes on "Guilt by Association" Analysis
Source: PLoS One. 2011 Feb 18;6(2):e17258. doi: 10.1371/journal.pone.0017258 (PMC3041792; doi:10.1371/journal.pone.0017258)

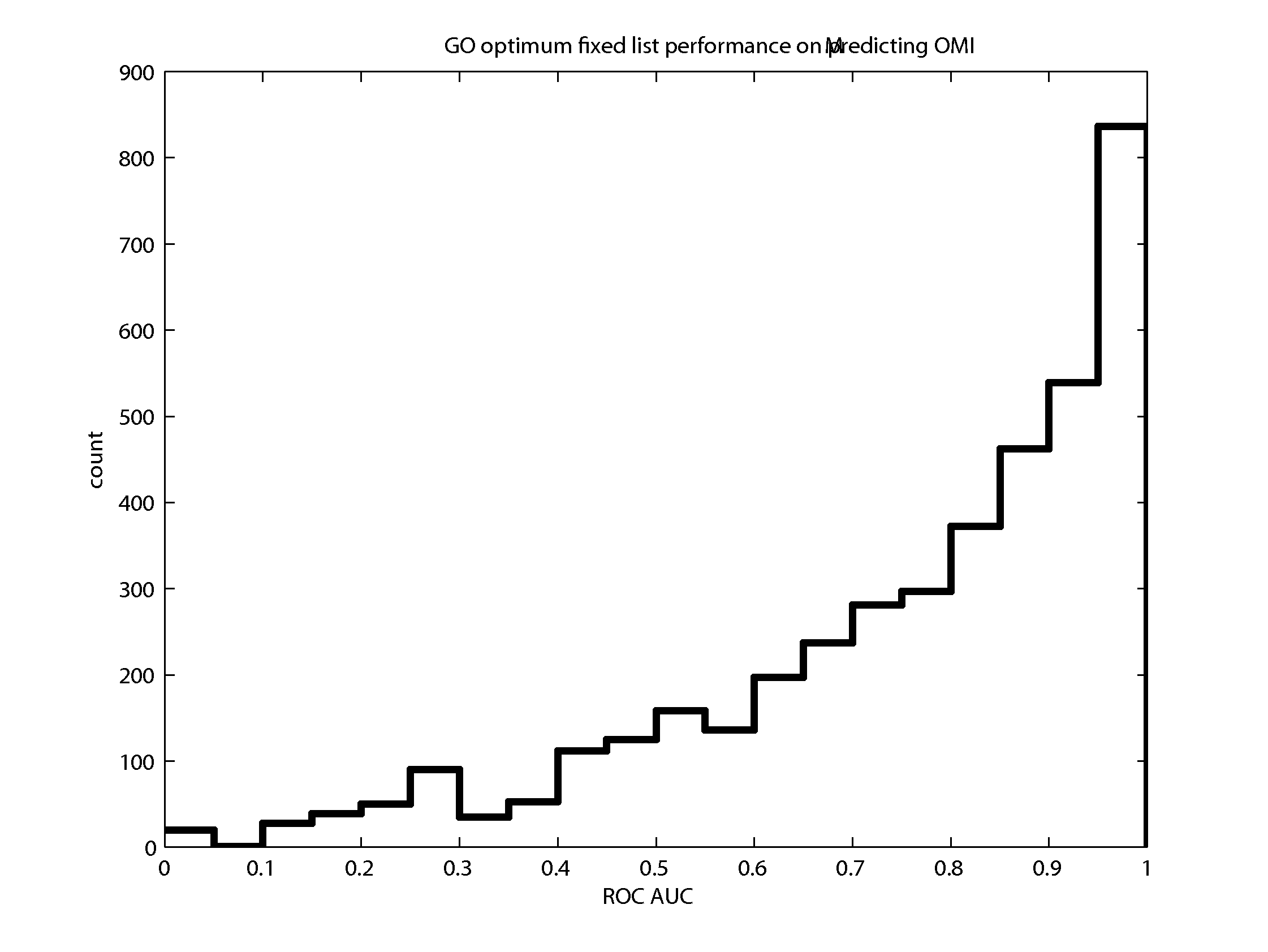

Supplement: Figure S1 — Predicting OMIM condition using the optimal gene ranking derived from GO. The distribution of AUC values is shown when predicted using the same optimal vector used in Figure 1A. (TIF) [file pone.0017258.s002.tif]

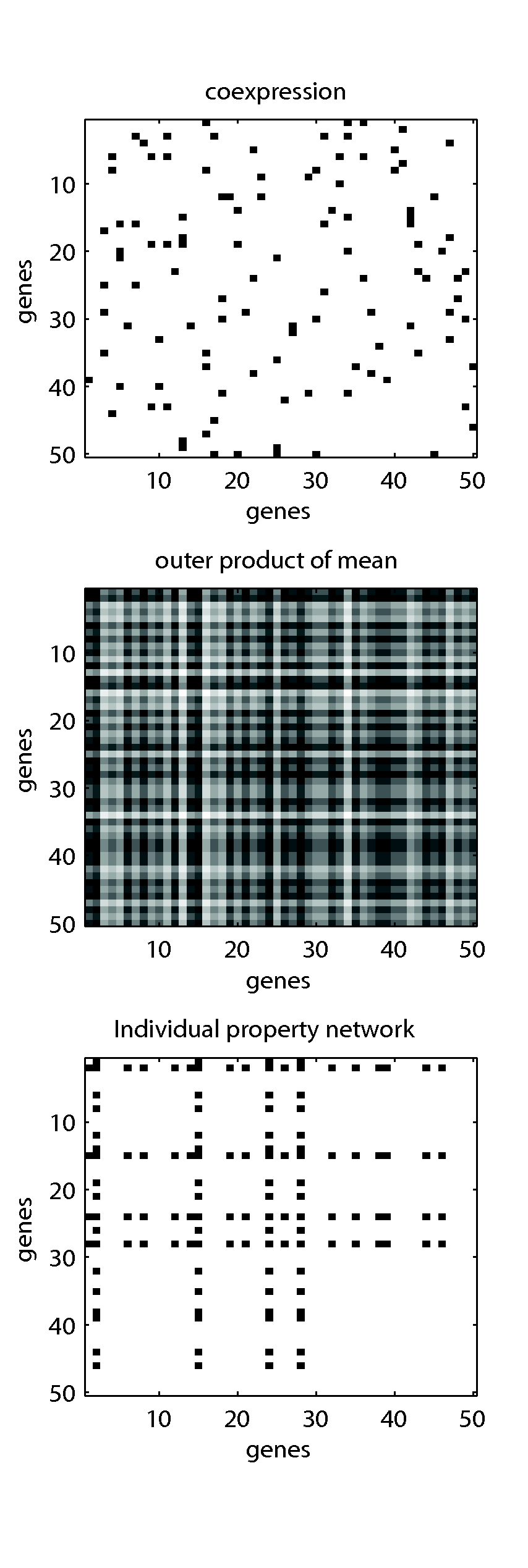

Supplement: Figure S2 — Schematic of construction of the Individual property network (IPN). Top: an original sparsified association matrix (black = association). Middle: Outer product of the Associability vector. Bottom: After processing the outer product to yield an “association matrix” of equivalent sparsity to the original data. (TIF) [file pone.0017258.s003.tif]

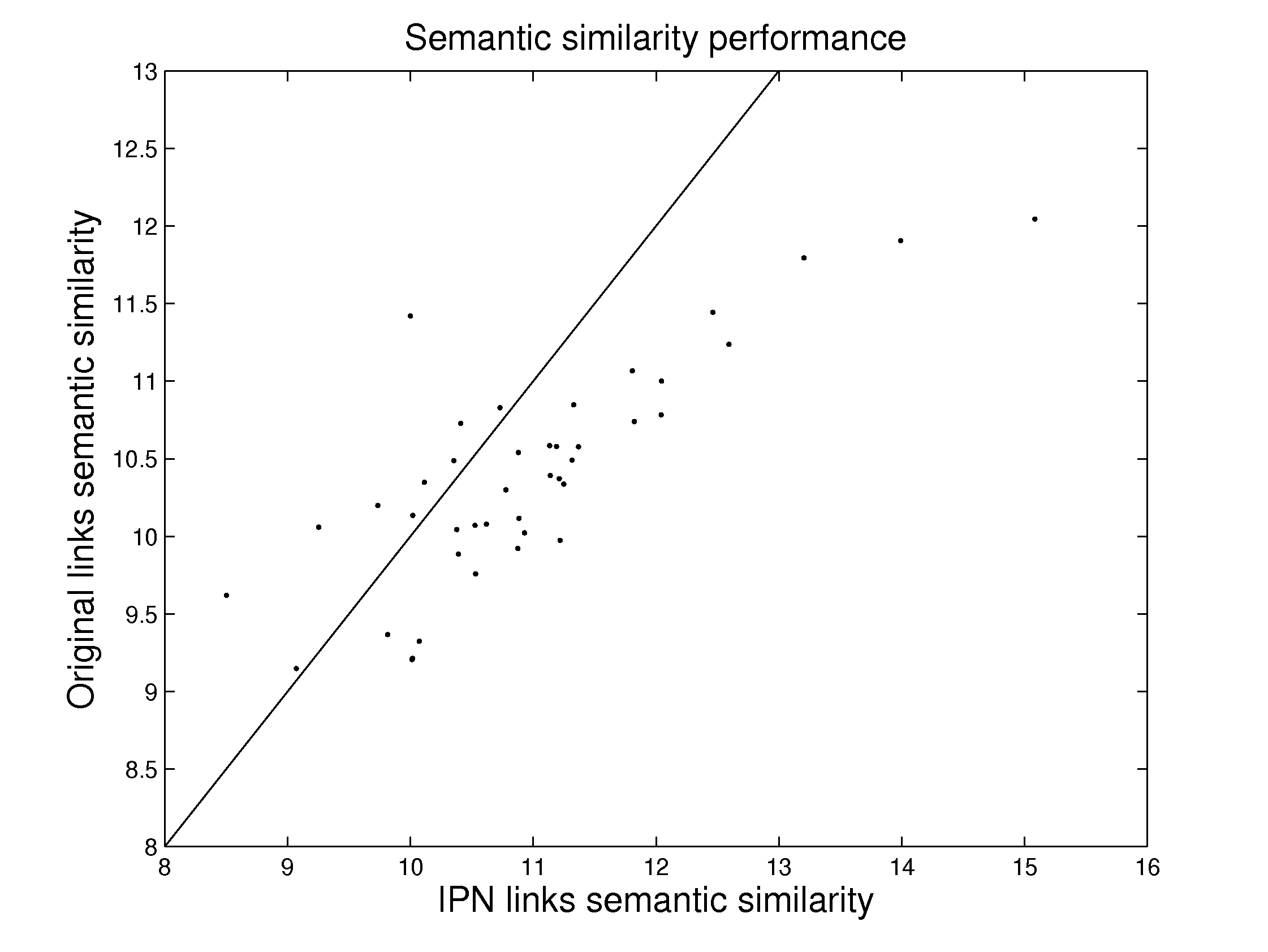

Supplement: Figure S3 — Semantic similarity. Semantic similarity allows us to assess common gene function in an association matrix without the use of a prediction algorithm. Using common GO term overlap, IPN performance is superior to original network performance. (TIF) [file pone.0017258.s004.tif]

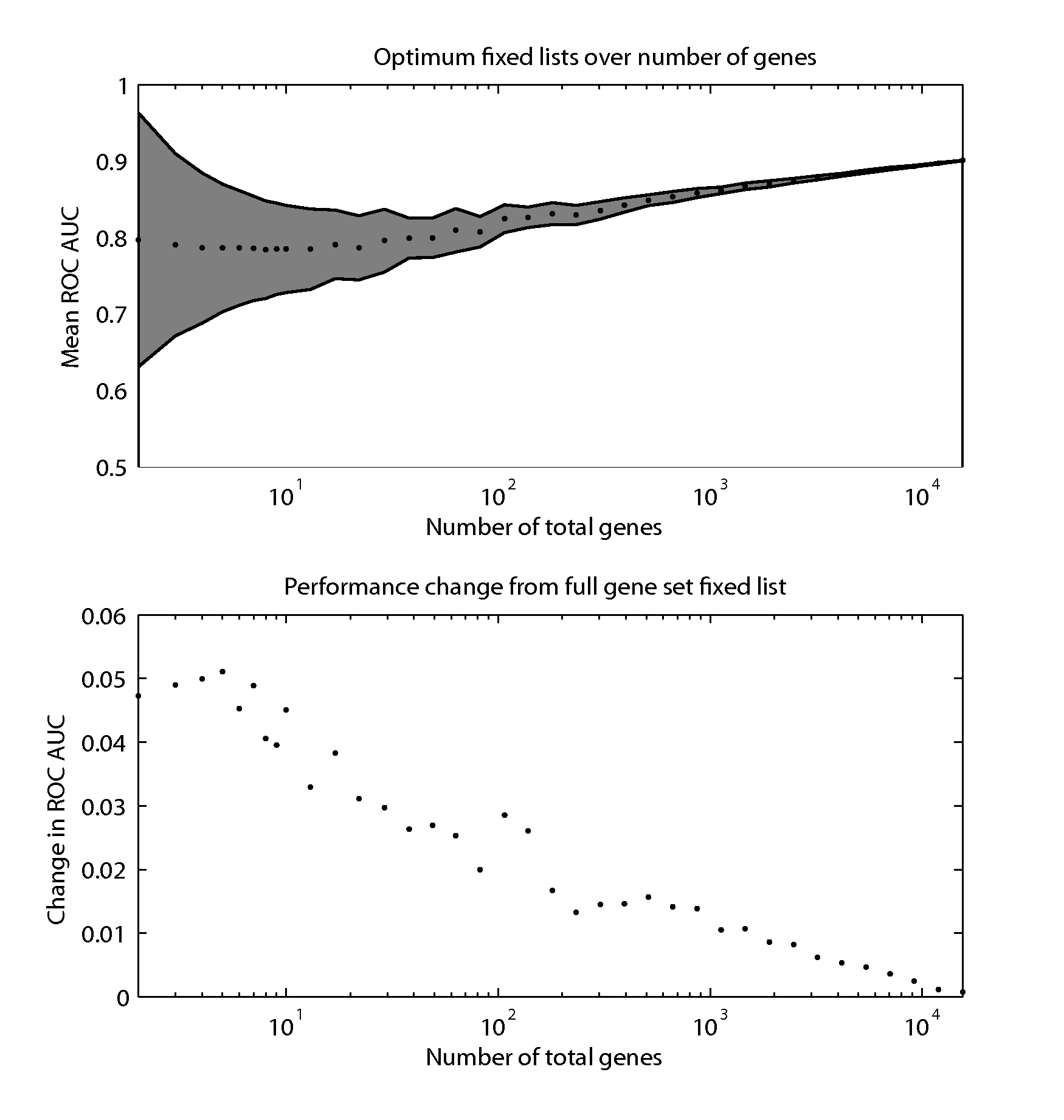

Supplement: Figure S4 — Performance of the optimal list is insensitive to using subsets of genes. Genes were randomly selected and the optimal list for those genes was generated. Top: The performance of the optimal list is strong even for very small groups of genes, with standard deviation shown by the grey region. Bottom: The error generated by using the optimal list over all genes is shown as a function of the number of genes included in analysis. Even for small groups, using the original optimal list is a reasonable approximation of constructing a specific list representing the subset of genes used. (TIF) [file pone.0017258.s005.tif]

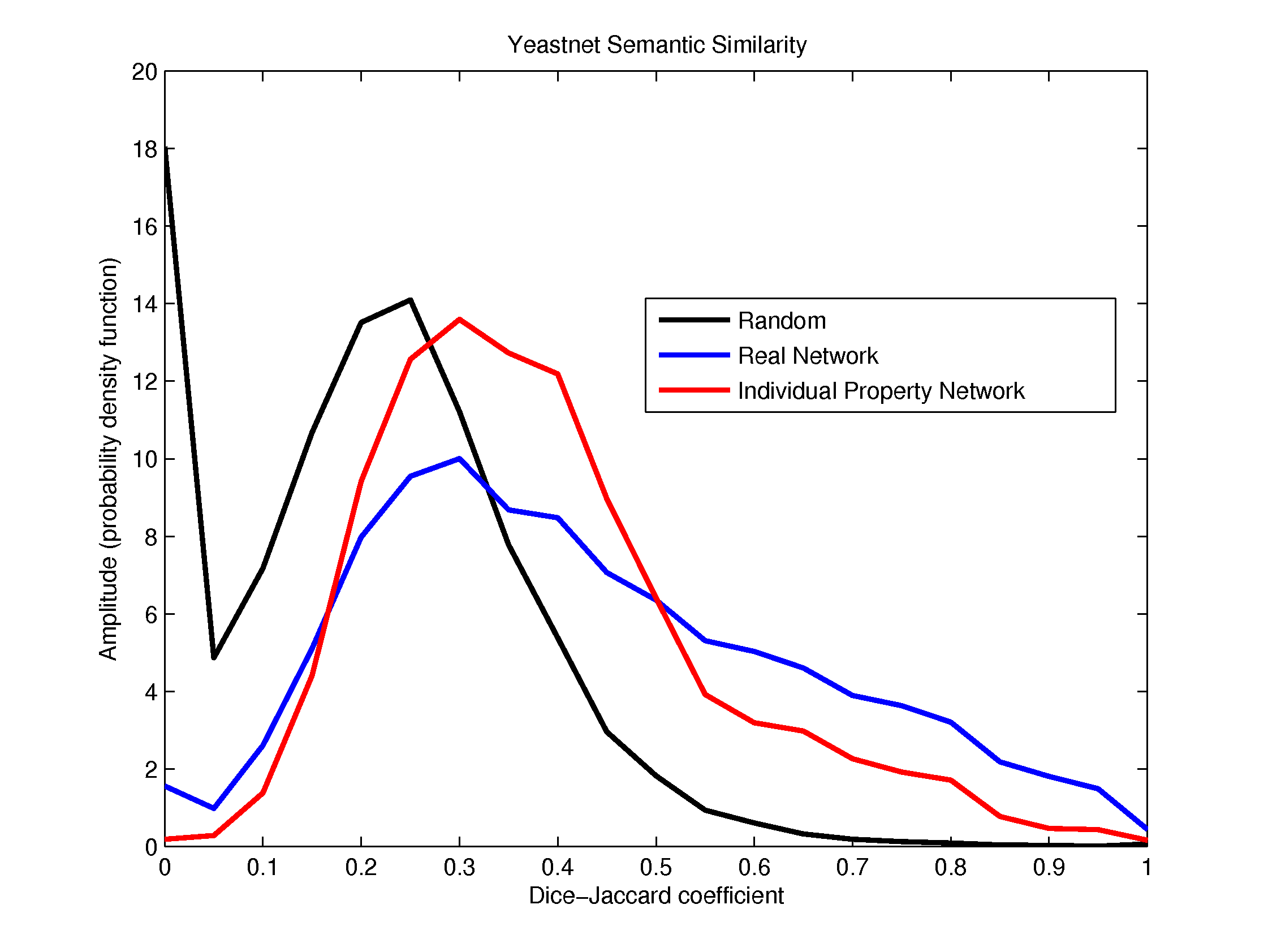

Supplement: Figure S5 — Performance as a function of number of genes. Gene function performance is plotted along with standard deviation with increasing GO size. (TIF) [file pone.0017258.s006.tif]

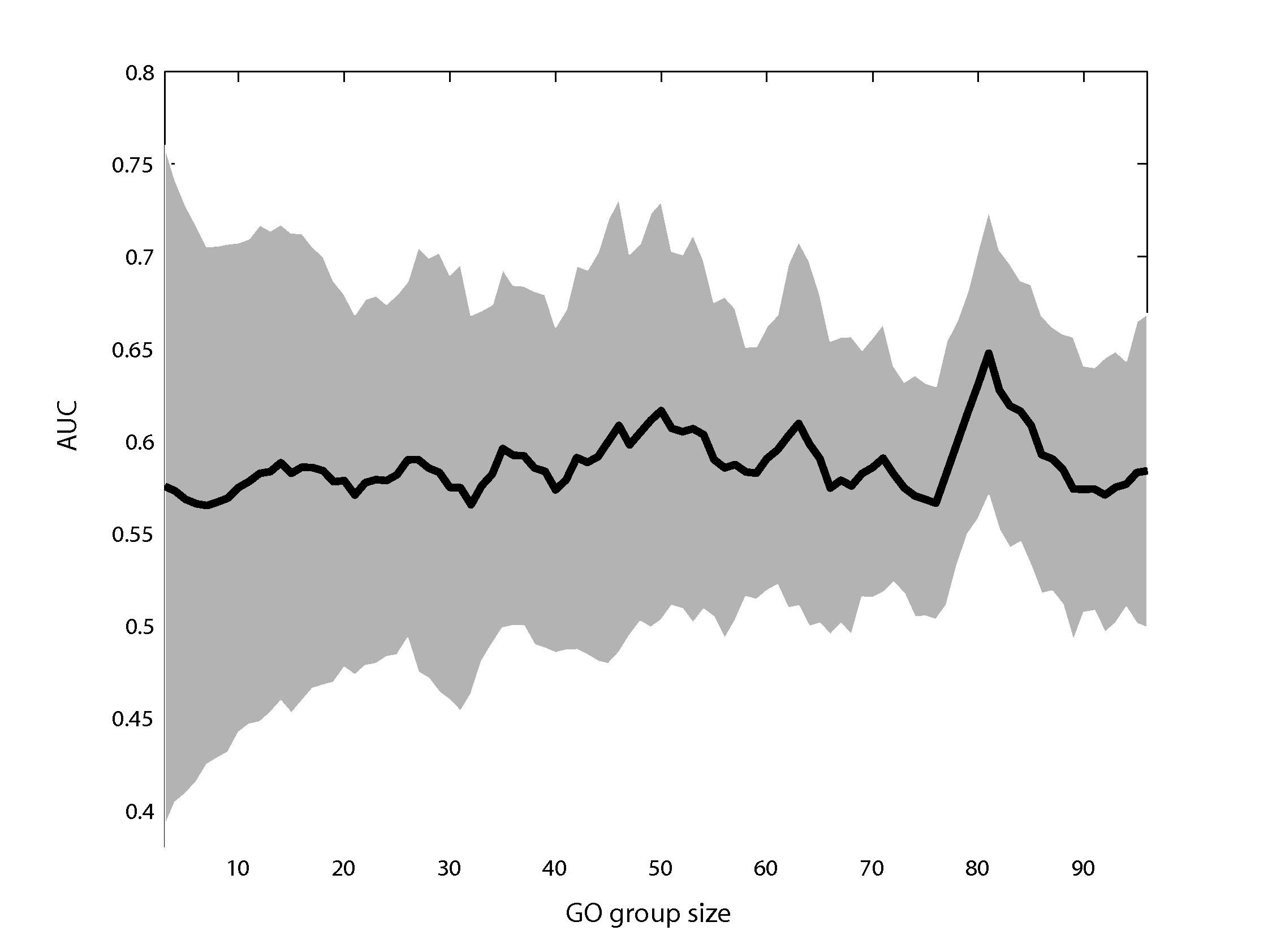

Supplement: Figure S6 — Yeastnet distribution of semantic similarities. The semantic similarity distribution over all links in the dataset is shown, as well as the similar distributions for random data and the Individual Property Network. (TIF) [file pone.0017258.s007.tif]
